# Decoding 2.3 million ECGs: interpretable deep learning for advancing cardiovascular diagnosis and mortality risk stratification

**DOI:** 10.1093/ehjdh/ztae014

**Published:** 2024-02-19

**Authors:** Lei Lu, Tingting Zhu, Antonio H Ribeiro, Lei Clifton, Erying Zhao, Jiandong Zhou, Antonio Luiz P Ribeiro, Yuan-Ting Zhang, David A Clifton

**Affiliations:** Institute of Biomedical Engineering, Department of Engineering Science, University of Oxford, Oxford, OX3 7DQ, UK; School of Life Course and Population Sciences, King’s College London, London, SE1 1UL, UK; Institute of Biomedical Engineering, Department of Engineering Science, University of Oxford, Oxford, OX3 7DQ, UK; Department of Information Technology, Uppsala University, Uppsala, Sweden; Nuffield Department of Population Health, University of Oxford Big Data Institute, Oxford, OX3 7LF, UK; Psychological Science and Health Management Center, Harbin Medical University, Harbin, 150076, China; Department of Psychiatry, University of Oxford, Oxford, OX3 7JX, UK; Department of Family Medicine and Primary Care, Li Ka Shing Faculty of Medicine, The University of Hong Kong, Hong Kong SAR, China; Division of Health Science, Warwick Medical School, University of Warwick, Coventry, CV4 7AL, UK; Department of Internal Medicine, Faculdade de Medicina, and Telehealth Center and Cardiology Service, Hospital das Clínicas, Universidade Federal de Minas Gerais, Belo Horizonte, Brazil; Department of Electronic Engineering, Chinese University of Hong Kong, Hong Kong SAR, China; Institute of Biomedical Engineering, Department of Engineering Science, University of Oxford, Oxford, OX3 7DQ, UK; Oxford Suzhou Centre for Advanced Research, Suzhou, 215123, China

**Keywords:** Interpretable diagnosis, Artificial intelligence, Electrocardiogram, Mortality risk stratification, Knowledge discovery

## Abstract

**Aims:**

Electrocardiogram (ECG) is widely considered the primary test for evaluating cardiovascular diseases. However, the use of artificial intelligence (AI) to advance these medical practices and learn new clinical insights from ECGs remains largely unexplored. We hypothesize that AI models with a specific design can provide fine-grained interpretation of ECGs to advance cardiovascular diagnosis, stratify mortality risks, and identify new clinically useful information.

**Methods and results:**

Utilizing a data set of 2 322 513 ECGs collected from 1 558 772 patients with 7 years follow-up, we developed a deep-learning model with state-of-the-art granularity for the interpretable diagnosis of cardiac abnormalities, gender identification, and hypertension screening solely from ECGs, which are then used to stratify the risk of mortality. The model achieved the area under the receiver operating characteristic curve (AUC) scores of 0.998 (95% confidence interval (CI), 0.995–0.999), 0.964 (95% CI, 0.963–0.965), and 0.839 (95% CI, 0.837–0.841) for the three diagnostic tasks separately. Using ECG-predicted results, we find high risks of mortality for subjects with sinus tachycardia (adjusted hazard ratio (HR) of 2.24, 1.96–2.57), and atrial fibrillation (adjusted HR of 2.22, 1.99–2.48). We further use salient morphologies produced by the deep-learning model to identify key ECG leads that achieved similar performance for the three diagnoses, and we find that the V1 ECG lead is important for hypertension screening and mortality risk stratification of hypertensive cohorts, with an AUC of 0.816 (0.814–0.818) and a univariate HR of 1.70 (1.61–1.79) for the two tasks separately.

**Conclusion:**

Using ECGs alone, our developed model showed cardiologist-level accuracy in interpretable cardiac diagnosis and the advancement in mortality risk stratification. In addition, it demonstrated the potential to facilitate clinical knowledge discovery for gender and hypertension detection which are not readily available.

## Graphical Abstract

**Graphical Abstract:**
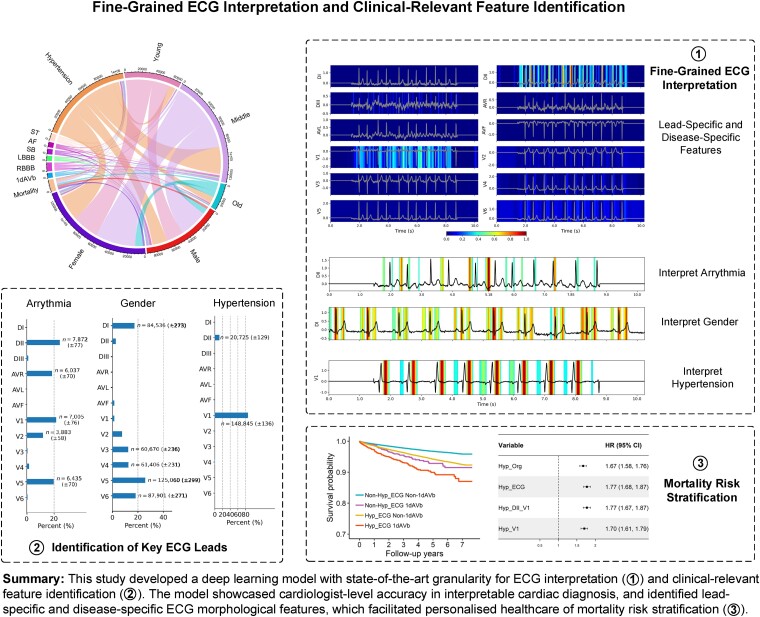

## Introduction

Electrocardiogram (ECG) is one of the most commonly performed cardiovascular diagnostic tests in cardiovascular medicine.^1^ The test provides an assessment of overall rhythm and cardiovascular status.^2,3^ Interpretation of ECGs is therefore critical to understand, diagnose, and stratify the risk of cardiovascular diseases (CVDs).^4^ However, the interpretation varies greatly, even among expert cardiologists; such variance between physicians presents a challenge to ensure consistency and reliability in ECG diagnosis. Meanwhile, the physician’s recognition of abnormal patterns is mostly limited to existing cardiac disorders, and it is, therefore, difficult to detect rare or relatively unknown diseases and recognize visually imperceptible elements in ECG morphology.

Artificial intelligence (AI) is rapidly emerging as a powerful tool in various healthcare applications.^3,5,6^ Artificial intelligence ECGs have been used to predict patients with refractory ventricular fibrillation,^7^ identify asymptomatic left ventricular dysfunction,^8^ and diagnose abnormality.^9^ While acknowledging the promise of AI-ECGs, previous studies indicated that explainability was a key limitation of many *deep* neural network (DNN) models.^10,11^ Most of these studies focused on improving model performance rather than extracting clinically useful information or expanding knowledge from ECG recordings. Identifying ECG features may offer novel findings that could provide new therapeutic targets or allow for clinicians to understand what drives the medical diagnosis. In addition, it is unreasonable for either the patient or the medical professional to accept an automated diagnosis at face value without justification.^12^ There are efforts to develop interpretable DNN models to produce explanations for ECG analysis, such as morphology segmentation^13^ and disease-specific feature visualization.^14^

Nevertheless, the interpretability has been a significant challenge in previous studies, attempting to extract meaningful features from ECG data.^13,14^ For instance, these studies often fail to identify important morphological features that have significant cardiac implications. They aimed to produce interpretable features from ECG recordings; however, the highlighted features included many unrelated ECG components and did not adequately capture the essential morphological features. More importantly, the visualized features in these studies had limited resolutions, making it difficult to explain the importance of ECG patterns precisely. In addition, the use of visualized ECG features to identify new medical information remains largely unexplored, such as identifying important ECG features and dominant leads for more efficient medical diagnosis. As highlighted by the PhysioNet/CinC Challenge,^15^ it is still challenging to identify reduced-lead ECGs that can capture the wide range of diagnostic information achieved by the 12-lead ECG recordings.

In this study, we hypothesize that AI models with a specific design can provide fine-grained interpretation of 12-lead ECGs for medical diagnosis, identify new clinically useful information, and that the ECG-predicted results can be further used to stratify the risk of mortality. To test this hypothesis, we created and trained an interpretable DNN model for a variety of medical diagnoses and mortality risk stratification using 2.3 million ECG recordings. We first validate the model by identifying and interpreting heart rhythm abnormalities. This is because arrhythmias are estimated to occur in 1.5–5% of the general population, making them one of the most prevalent heart disorders.^16^ In particular, arrhythmia, such as atrial fibrillation (AF), is a major public health concern associated with significant morbidity, mortality, and healthcare costs, affecting more than 37 million individuals globally.^17^ Other than the diagnosis of heart conditions, we test the developed DNN model in a more general task of gender identification. This is highly relevant to our central task, because gender differences have been observed in the development of CVDs and the risk of mortality. For example, women tend to develop heart diseases later in life than men, while also having worse outcomes and higher mortality.^18^ In a further step, we perform the third task of hypertension screening to validate the developed model in a wider context of medical practice. Hypertension is the largest single contributor to CVDs causing morbidity and mortality, with a rising prevalence affecting approximately 1.39 billion people worldwide.^19^ The ECG-predicted results are then used to stratify the risk of mortality for cohorts with heart rhythm abnormalities, gender differences, and hypertension. To the best of our knowledge, this is the first time that an explanatory DNN model with fine granularity has been extensively investigated with such a sheer scale ECG data set. In particular, we identify fine-grained ECG features from deep learning, which are then used to derive reduced-ECG leads for a variety of medical diagnostic tasks.

## Methods

### Population and study design

**Figure 1:**
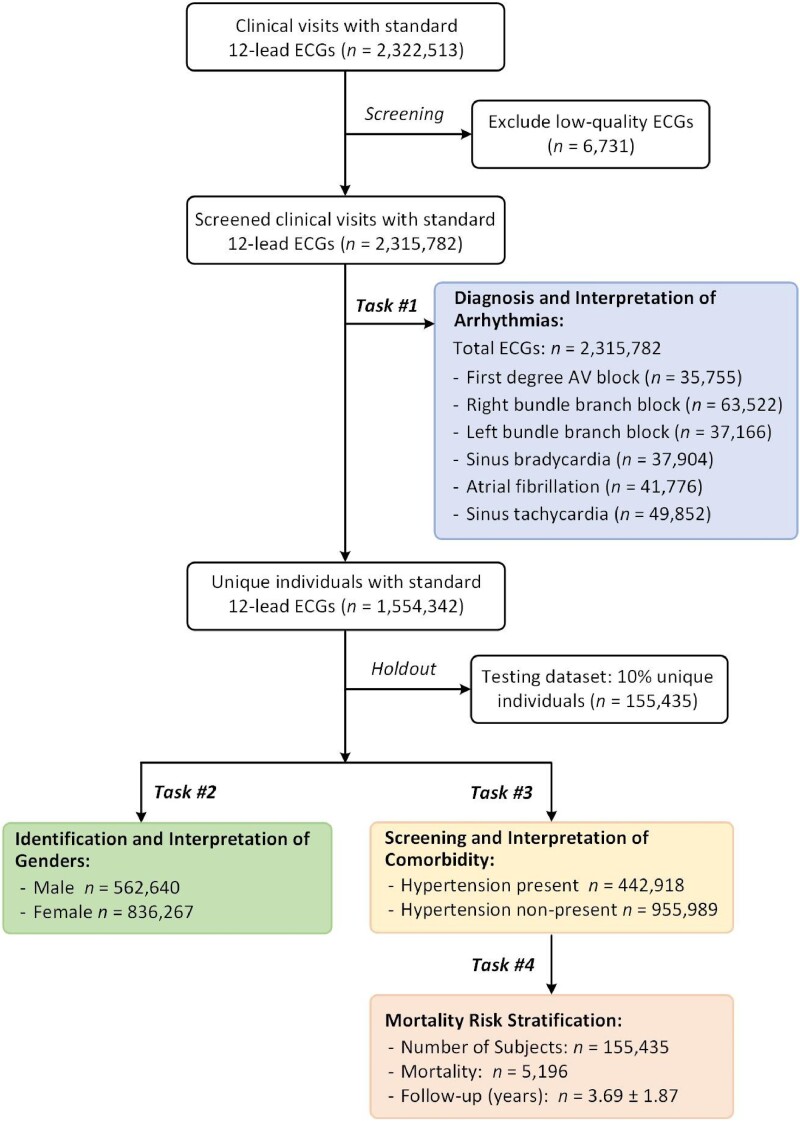
Dataflow and population characteristics for the three medical tasks in this study. The data were collected in primary care facilities with population characteristics as follows. (i) For the first task (Task #1), the electrocardiogram recordings were collected from a population with an average age of 53.64 *±* 17.42 years , and 60.26% were females (*n*_Female_ = 1 395 461). (ii) For the second task (Task #2), the population had an average age of 51.66 *±* 17.58  years and 59.78% were females (*n*_Female_ = 836 267). (iii) For the third task (Task #3), subjects with hypertension accounted for 31.66% of the whole population; the hypertension group had an average age of 59.33 *±* 14.79 years, and 62.71% were females (*n*_Female_ = 277 756). (iv) For the fourth task (Task #4), the cohort had a total of 155 435 subjects with 5196 death records (mortality rate: 3.34%), and had 7 years follow-up with a mean value of 3.69 years and the standard deviation of 1.87 years. We performed the diagnostic Tasks #1–#3 independently, and then, we implemented Task #4 for the hypertension cohort using electrocardiogram-predicted results. Detailed descriptions of the data set and population characteristics can be found in Supplementary material online, *Tables S1* and *S2*, and *Figure S2*.

The study retrieved a data set of 2 322 513 ECG recordings from 1 558 772 patients through the Telehealth Network of Minas Gerais, Brazil.^20^ The recordings were mostly collected during patients’ clinic visits to primary care facilities with follow-ups from 2010 to 2016, and the data were sampled with frequency rates from 300 to 600 Hz for a duration of 7–10 s (see Supplementary material online, *Section SA1*). As demonstrated in *Figure 1* and Supplementary material online, *Tables S1* and *S2*, we show the dataflow and study population for the four medical tasks in this study. (i) In the first task, we use 2 315 782 ECG recordings to train the DNN model for the diagnosis of cardiac abnormalities, including the first-degree atrioventricular block (1dAVb), right bundle branch block (RBBB), left bundle branch block (LBBB), sinus bradycardia (SB), AF, and sinus tachycardia (ST). The trained DNN model is then tested on an unseen ECG data set, which is rigorously annotated by cardiologists. (ii) In the second task, we train the DNN model for gender identification using ECGs collected from 1 398 907 subjects (female: 59.78%, *n*_Female_ = 836 267), which is tested with a holdout ECG data set sampled from 155 435 subjects (female: 59.52%, *n*_Female_ = 92 513). (iii) In the third task, the model is trained to screen hypertension for 1 398 907 subjects (hypertension: 31.66%, *n*_Hypertension_ = 442 918), and tested with 155 435 subjects (hypertension: 31.65%, *n*_Hypertension_ = 49 202). (iv) In the fourth task, we use the ECG-predicted results to stratify the risk of mortality considering cardiac abnormalities, gender differences, and hypertension; and the holdout cohort (*n*_Subjects_ = 155 435) has a mortality rate of 3.34% within the 7 follow-up years.

### Model development

We developed an interpretable DNN model for ECG interpretation with fine granularity, based on a novel *isolation-integration* strategy for feature visualization (see Supplementary material online, *Section SA2*). The architecture of our developed DNN model is illustrated in Supplementary material online, *Figure S1*. For the identification of salient morphologies using the DNN model, the ECG data are first processed using our proposed isolation strategy, then we integrate the ECG features from all leads and use them for the model prediction; Next, we compute the gradient for the predicted output with respect to the concatenated feature matrix, and use the averaged gradient scores to weight kernels, followed by a ReLU function to filter the positive values. By performing dimension alignment between the concatenated feature matrix and the ECG data, a feature heatmap can be obtained to weigh the data importance in the ECG recording. Notably, the concatenated feature matrix is computed using an isolated strategy, which enables learning data importance for each ECG lead precisely rather than shared weights across different leads. The concatenated feature matrix has the half-length of the input ECG recording in the temporal direction, and the calculated heatmap only needs to be magnified two times for the dimension alignment. Thus, our developed DNN model allows producing salient features for fine-grained ECG interpretation.

## Results

### Diagnosis and interpretation of electrocardiogram abnormalities

**Figure 2:**
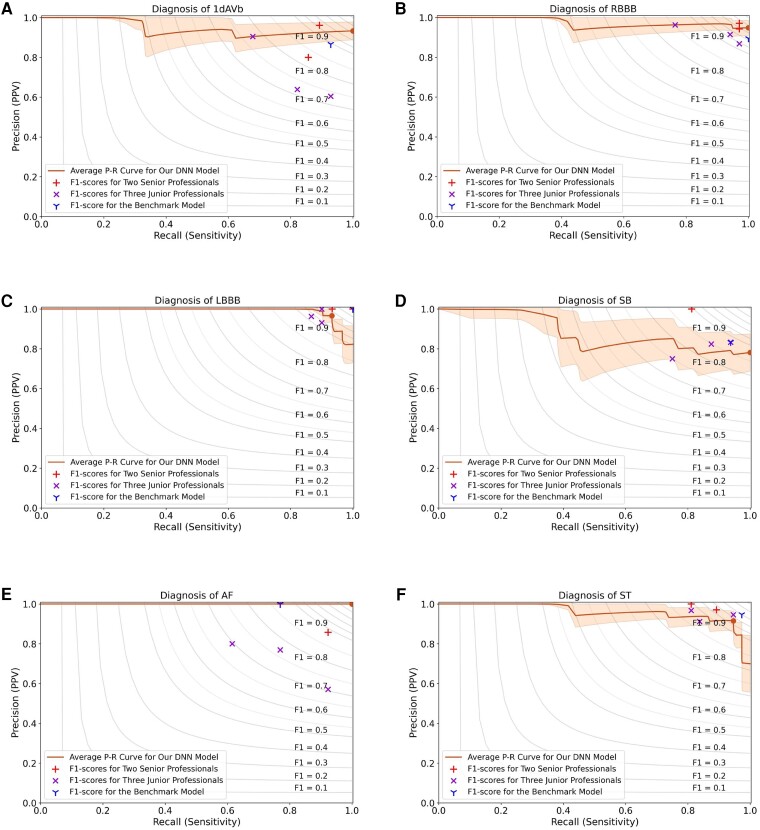
Performance comparison for the diagnosis of abnormalities, including (*A*) first-degree atrioventricular block, (*B*) right bundle branch block, (*C*) left bundle branch block, (*D*) sinus bradycardia, (*E*) atrial fibrillation, and (*F*) sinus tachycardia. This figure shows the precision-recall curves for the performance of our DNN model, evaluation results from five cardiology professionals, and the result of the benchmark model.^21^ The solid lines are the average precision-recall curves for the diagnosis of arrhythmias, and the shading areas represent standard deviations obtained by the bootstrap method. The circle dots correspond to *F*_1_ scores for our DNN model, the ‘+’ symbols are used to denote *F*_1_ scores for the two senior professionals, ‘X’ for the three junior professionals, and ‘Y’ for the benchmark DNN model. The contour plots show the iso-*F*_1_ curves with a constant value for each curve, and a point closes to the ideal score of ‘1’ in the top-right corner indicating a higher *F*_1_ score.

In the first task, the DNN model has a micro-average area under the receiver operating characteristic curve (AUC) score of 0.998 [95% confidence interval (CI), 0.995–0.999] and an *F*_1_ score of 0.948 (95% CI, 0.921–0.971) on identifying the ECG abnormalities. We compare the performance of our DNN model with the evaluation results from three junior professionals with experience in ECGs, two senior cardiologists, and the state-of-the-art study.^21^ It can be seen from *Table 1* and *Figure 2* that the highest evaluation score from the three junior professionals is 0.876 (95% CI, 0.830–0.915); the two senior cardiologists have the highest *F*_1_ score of 0.945 (95% CI, 0.914–0.970); and the state-of-the-art benchmark has a score of 0.938 (95% CI, 0.910–0.961).^21^ Compared with the evaluation results yielded by the cardiology professionals, our DNN model has better performance than the three junior professionals and one senior professional in the diagnosis of 1dAVb (*P* = 0.043, Supplementary material online, *Table S4*), and outperforms the diagnostic performance of AF from three junior professionals (*P* = 0.041).

**Table 1:** Performance comparison for the diagnosis of abnormalities using standard 12-lead electrocardiogram recordings

	*F*_1_ score (95% CI)						
	Junior professionals			Senior professionals		DNN models	
	Cardio. Rd.	Emerg. Rd.	Medical Sd.	Cardio. #1	Cardio. #2	A.H.R.^21^	Our DNN
1dAVb	0.776 (0.625–0.889)	0.719 (0.578–0.831)	0.732 (0.605–0.836)	0.828 (0.704–0.925)	0.926 (0.844–1.000)	0.897 (0.793–0.969)	0.966 (0.912–1.000)
RBBB	0.917 (0.842–0.974)	0.852 (0.746–0.939)	0.928 (0.852–0.985)	0.957 (0.899–1.000)	0.971 (0.921–1.000)	0.944 (0.881–0.989)	0.971 (0.928–1.000)
LBBB	0.947 (0.875–1.000)	0.912 (0.828–0.980)	0.915 (0.830–0.983)	0.966 (0.907–1.000)	1.000 (1.000–1.000)	1.000 (1.000–1.000)	0.949 (0.882–1.000)
SB	0.882 (0.743–0.976)	0.848 (0.692–0.963)	0.750 (0.538–0.889)	0.897 (0.750–1.000)	0.897 (0.741–1.000)	0.882 (0.750–0.976)	0.865 (0.727–0.973)
AF	0.769 (0.545–0.933)	0.696 (0.400–0.875)	0.706 (0.500–0.865)	0.870 (0.667–1.000)	0.889 (0.737–1.000)	0.870 (0.667–1.000)	1.000 (1.000–1.000)
ST	0.882 (0.789–0.951)	0.946 (0.881–0.989)	0.873 (0.778–0.949)	0.896 (0.813–0.965)	0.930 (0.853–0.987)	0.960 (0.904–1.000)	0.933 (0.862–0.987)
Micro-avg	**0.876 (0.830–0.915)**	0.846 (0.793–0.892)	0.833 (0.789–0.876)	0.908 (0.871–0.941)	**0.945 (0.914–0.970)**	0.938 (0.910–0.961)	**0.948 (0.921–0.971)**

The bold-faced scores denote the best performance for the three junior professionals, two senior professionals, and two deep-learning models.

Cardio. Rd., 4th year cardiology residents; Emerg. Rd., 3rd year emergency residents; Medical Sd., 5th year medical students. Cardio. #1, the 1st cardiologist; Cardio. #2, the 2nd cardiologist; A.H.R., the state-of-the-art benchmark model developed in^21^; DNN, the deep-learning model developed in this study.

**Figure 3:**
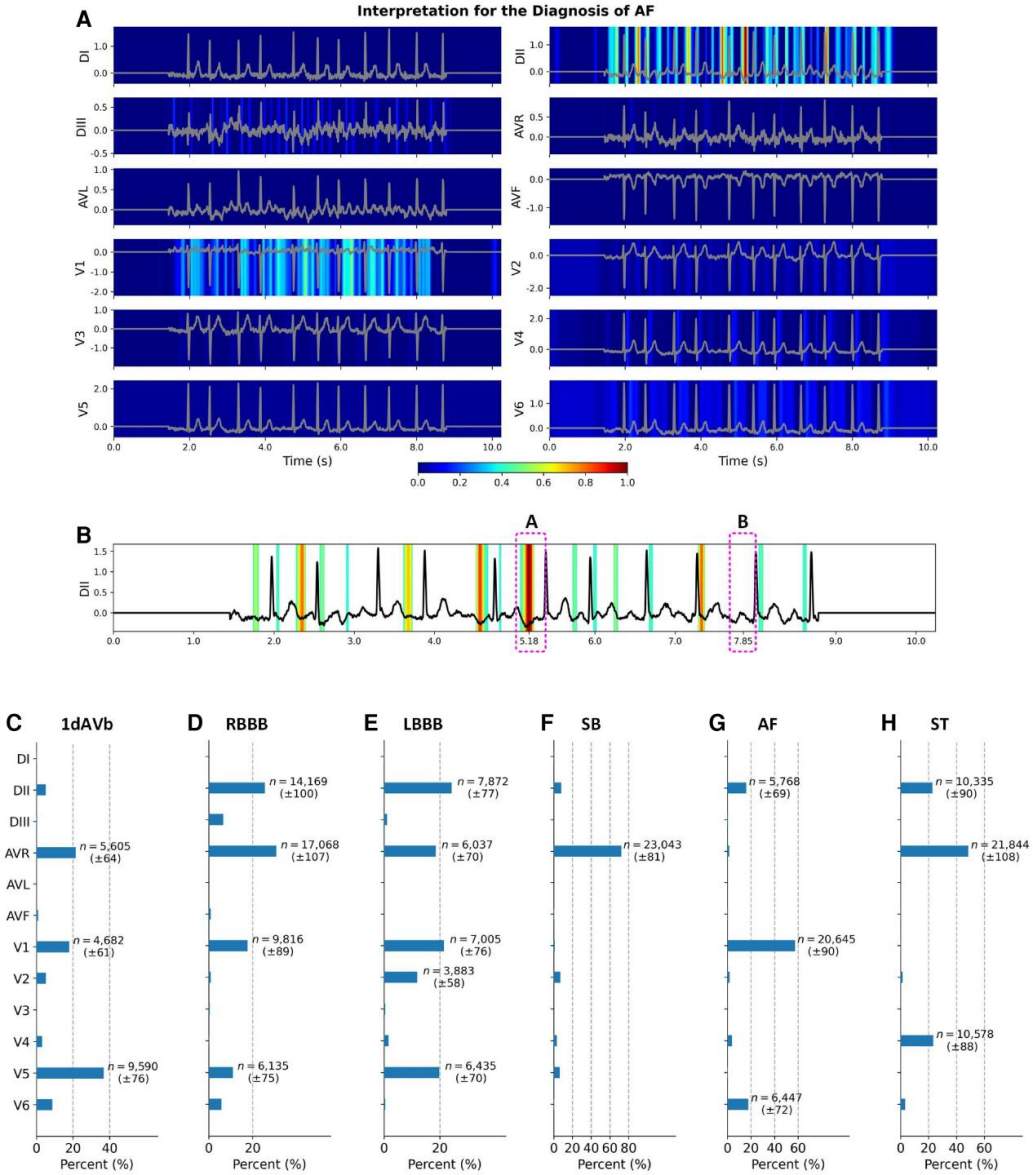
Interpretation for the diagnosis of atrial fibrillation and distributions of dominant electrocardiogram leads. (*A*) The original calculated heatmaps for the diagnosis of atrial fibrillation using 12 electrocardiogram leads, with colour bar ranging from blue to red indicating the increasing weights of data importance. (*B*) The refined view of the DII lead in (*A*) by removing background colours with values <0.4. Segments *A* and *B* show the inconsistent morphologies in the locations of P waves in the DII lead. We show distributions of dominant electrocardiogram leads for the diagnosis of (*C*) first-degree atrioventricular block, (*D*) right bundle branch block, (*E*) left bundle branch block, (*F*) sinus bradycardia, (*G*) atrial fibrillation, and (*H*) sinus tachycardia. We annotate the number of occurrences when the dominant lead accounts for >10% of all the 12 electrocardiogram leads. The number of occurrences is presented as mean and standard deviation calculated by the bootstrap method.^21^.

The diagnosis of AF particularly has important medical implications, which is a leading cardiac cause of stroke, heart failure, and mortality.^22^ It can be seen from *Table 1* that among all the five professionals, the highest *F*_1_ score for the diagnosis of AF is 0.889 (95% CI, 0.737–1.000); and the benchmark model also has moderate performance with a score of 0.870 (95% CI, 0.667–1.000).^21^ In contrast, our developed DNN model successfully identifies all AF in the data set. To interpret the diagnosis by the DNN model, we calculate the heatmap with fine-grained resolution for each of the 12 ECG leads, and highlight the salient information that has been used for the decision-making. It can be seen from *Figure 3A* that the DNN model uses salient information in the DII and V1 leads for the diagnosis, and has the most important features in the DII lead.

Generally, the absence of P waves in an ECG recording can be used to diagnose AF. However, artefacts or fibrillatory waves can mimic P waves and lead to misdiagnosis.^23^*Figure 3B* shows the fine-grained interpretation of the DII lead produced by our DNN models. It can be seen from *Figure 3B* that the P wave is absent in some areas of the ECG morphology, e.g. segment *A* (around 5.18 s); and there are also waves clearly presented in some areas, e.g. segment *B* (around 7.85 s). The inconsistent morphologies in the locations of P waves challenge the accurate diagnosis of AF. In contrast, our developed DNN model is flexible in identifying important features, and it highlights the importance of segment *A* rather than segment *B*, which is consistent with existing diagnostic criteria.^23^ As well as identifying the absence of P waves, the DNN model also recognizes S waves as important features in the DII lead, and other features in the V1 lead. Combining salient information from different ECG leads, our DNN model makes a comprehensive decision with a prediction probability of 0.961 for the diagnosis.

Other than interpreting the diagnosis of AF, we provide the interpretation of diagnosing all other types of ECG abnormalities in Supplementary material online, *Figures S3–S7*. In a further step, we derive dominant ECG leads that are derived from the identified salient features. We first filter ECG recordings in the whole data set with a prediction probability higher than 0.8, which indicates the DNN model having confident outputs for the diagnosis. Then, we sum values of the heatmap for each ECG lead, and identify the dominant lead with the highest value for the ECG recording. To show distributions of the identified dominant leads, we calculate their occurrences and percentages among all the 12 ECG leads. It can be seen from *Figure 3C–H* that the ECG abnormalities have varied distributions of dominant leads; the 1dAVb has AVR, V1, and V5 as dominant leads; both the RBBB and LBBB have dominant leads of DII, AVR, V1, and V5; the SB has a prominent AVR lead; the AF has three dominant leads of DII, V1 and V6; and the ST has dominant DII, AVR and V4 leads. We investigate the effectiveness of our identified dominant leads in the diagnosis of ECG abnormalities. As shown in *Figure 3C–H*, the AVR and V1 leads are two representative leads for the ECG abnormalities. Using the two ECG leads, as shown in *Table 2*, our DNN model achieves an AUC score of 0.990 (95% CI, 0.982–0.995) and an *F*_1_ score of 0.879 (95% CI, 0.834–0.919), which is comparable with the best performance of the three junior professionals (*P* = 0.505).

**Table 2:** Performance comparison for the diagnosis of abnormalities using electrocardiogram recordings with dominant leads

	Dominant AVR and V1 leads			Dominant DII, AVR, and V1 leads		
	Precision (95% CI)	AUC (95% CI)	*F*_1_ score (95% CI)	Precision (95% CI)	AUC (95% CI)	*F*_1_ score (95% CI)
1dAVb	0.870 (0.714–1.000)	0.991 (0.982–0.998)	0.784 (0.632–0.898)	0.952 (0.842–1.000)	0.992 (0.982–0.998)	0.816 (0.667–0.927)
RBBB	0.935 (0.833–1.000)	0.996 (0.992–0.999)	0.892 (0.800–0.964)	0.909 (0.800–1.000)	0.997 (0.993–0.999)	0.896 (0.807–0.966)
LBBB	0.966 (0.889–1.000)	0.978 (0.931–1.000)	0.949 (0.875–1.000)	1.000 (1.000–1.000)	0.993 (0.982–1.000)	0.947 (0.880–1.000)
SB	0.762 (0.565–0.947)	0.998 (0.995–1.000)	0.865 (0.722–0.973)	0.842 (0.647–1.000)	0.999 (0.996–1.000)	0.914 (0.786–1.000)
AF	0.857 (0.636–1.000)	0.998 (0.993–1.000)	0.889 (0.727–1.000)	1.000 (1.000–1.000)	0.997 (0.992–1.000)	0.917 (0.750–1.000)
ST	0.868 (0.750–0.972)	0.997 (0.993–0.999)	0.880 (0.786–0.956)	0.878 (0.757–0.973)	0.998 (0.995–1.000)	0.923 (0.844–0.976)
Micro-avg	0.885 (0.830–0.937)	0.990 (0.982–0.995)	**0.879 (0.834–0.919)**	0.921 (0.875–0.962)	0.995 (0.992–0.997)	**0.903 (0.868–0.935)**

The term ‘Dominant AVR and V1 leads’ indicates that the model has inputs with only two ECG leads, e.g. AVR and V1 leads, rather than using 12 ECG leads. The bold-faced values highlight the model performance of micor average *F*_1_ scores using dominant ECG leads.

Additionally, we validate our developed DNN model on an external data set, which is retrieved from the PhysioNet/CinC Challenge 2017.^24^ The DNN model was trained using *n*_ECGs_ = 8528 ECG recordings, and tested on a holdout validation data set. Supplementary material online, *Table S6* presents the model performance for classifying the different types of ECG recordings. It can be seen from Supplementary material online, *Table S6* that the DNN model has a micro-average *F*_1_ score of 0.884, precision score of 0.894, and recall score of 0.873 for the identification. In particular, our DNN model has an *F*_1_ score of 0.929 on the diagnosis of AF and 0.921 on classifying noise signals. The results indicate that despite the widespread noises in ECG recordings, our model demonstrates robust performance in the diagnosis of heart rhythm abnormalities.

### Identification and interpretation of genders

**Figure 4:**
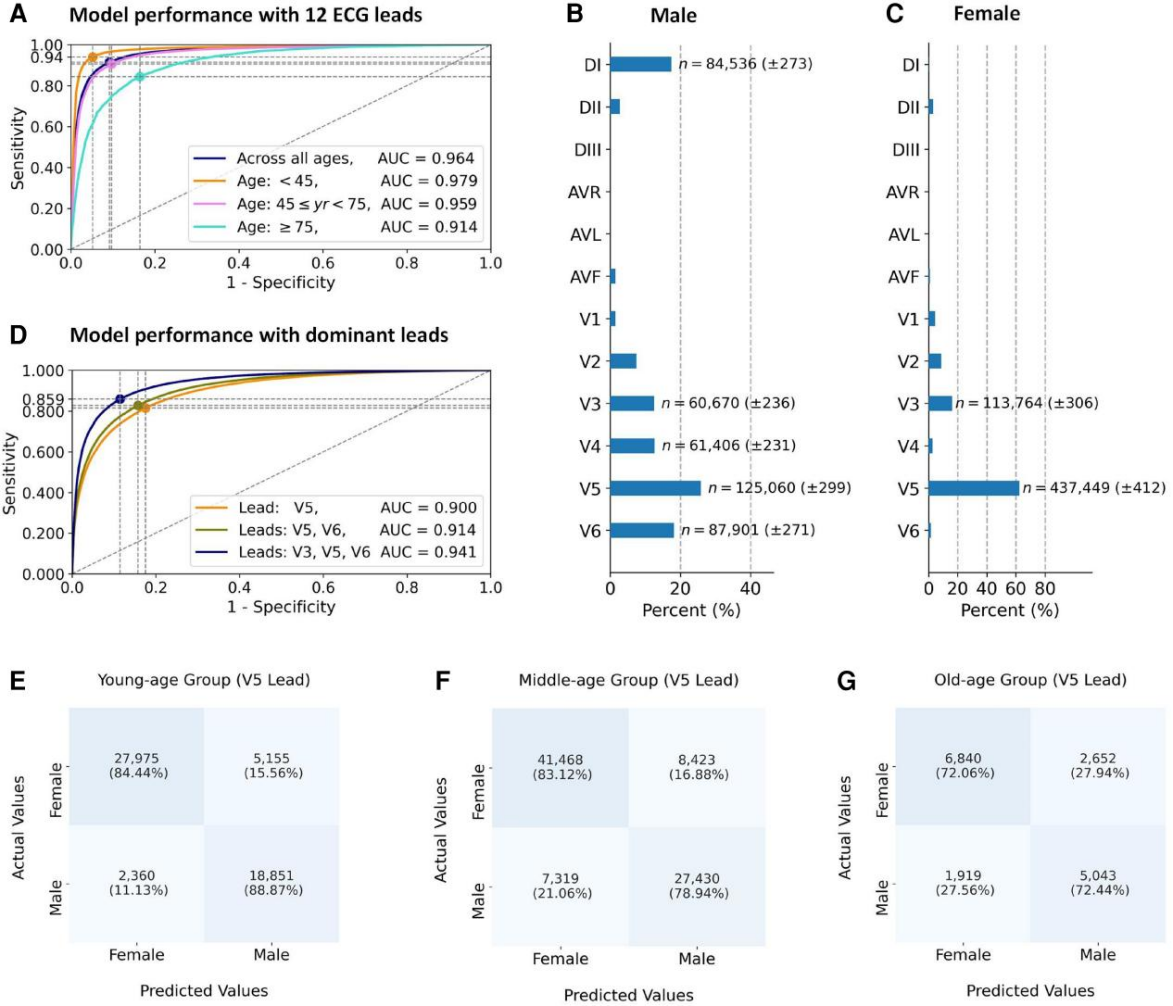
Model performance and lead importance for gender identification using our proposed DNN model. (*A*) Performance comparison of the DNN model for gender identification using 12-lead electrocardiogram recordings in different age groups. (*B*) Distributions of dominant leads for identifying male subjects. (*C*) Distribution of dominant leads for identifying female subjects. (*D*) Performance comparison between different dominant electrocardiogram leads. We demonstrate confusion matrices for gender identification using the dominant V5 lead in different age groups, including (*E*) the young-age group (*<*45 years), (*F*) the middle-age group (≥45 and *<*75 years), and (*G*) the old-age group (*≥*75 years). In each of the receiver operating characteristic curves, the dot point indicates the optimal cut-off point for the sensitivity and specificity calculated by the G-mean method.^25^

In the second task, we use the DNN model to identify genders for individual subjects (*n*_Subjects_ = 155 435), and our DNN model has an AUC score of 0.964 (95% CI, 0.963–0.965) for gender identification (*Figure 4*). As ECG features change over time due to normal ageing,^26^ we investigate the model performance in different age groups, i.e. young-age [years (yr) *<* 45, *n*_Subjects_ = 54 341], middle-age (45 *≤* yr *<* 75, *n*_Subjects_ = 84 640), and old-age groups (yr *≥* 75, *n*_Subjects_ = 16 454).^27^ It can be seen from *Figure 4A* that our DNN model has an AUC score of 0.979 (95% CI, 0.977–0.980) on identifying genders for the young-age group, which is higher than the AUC score of 0.959 (95% CI, 0.958–0.961) for the middle-age group, and 0.914 (95% CI, 0.909–0.918) for the old-age group.

We show salient features in ECG recordings for the interpretation of gender identification in Supplementary material online, *Figures S9* and *S10*. It can be seen from Supplementary material online, *Figure S9* that the DNN model mainly uses salient features from the DII, V1, and V5 leads for identifying the female subject. For identifying the male subject, the model uses the DI, V4, V5, and V6 leads. We then analyse the distribution of dominant leads for gender identification using the method as presented in the first task. *Figure 4B* and *C* present distributions of dominant leads for identifying male and female subjects separately. It can be seen from *Figure 4B* and *C* that V5 is the most used ECG lead for gender identification by the DNN model, which is a dominant lead for identifying male subjects (*n*_Male_ = 125 060 *±* 299) and female subjects (*n*_Female_ = 437 449 *±* 412). Other than the V5 lead, V3 also appears as a dominant ECG lead for identifying the male subjects (*n*_Male_ = 60 670 *±* 236) and female subjects (*n*_Female_ = 113 764 *±* 306).

In addition, we present detailed comparisons of model performance using the identified dominant ECG leads for gender identification in Supplementary material online, *Figures S13–S15* and *Tables S7–S9*. The results show that using DI, V3, and V5 leads, the DNN model has the highest performance (*P <* 0.01) with an AUC score of 0.970 (95% CI, 0.969–0.972) and a diagnostic odds ratio (DOR) of 145.891 (95% CI, 139.089–156.331) for gender identification. We note that all models have higher performance in identifying genders in the young-age group than in the old-age group (*P <* 0.01), which have the highest AUC score of 0.885 (95% CI, 0.880–0.890) in the old-age group using the DI, V3, and V5 leads.

### Screening and interpretation of hypertension

**Figure 5:**
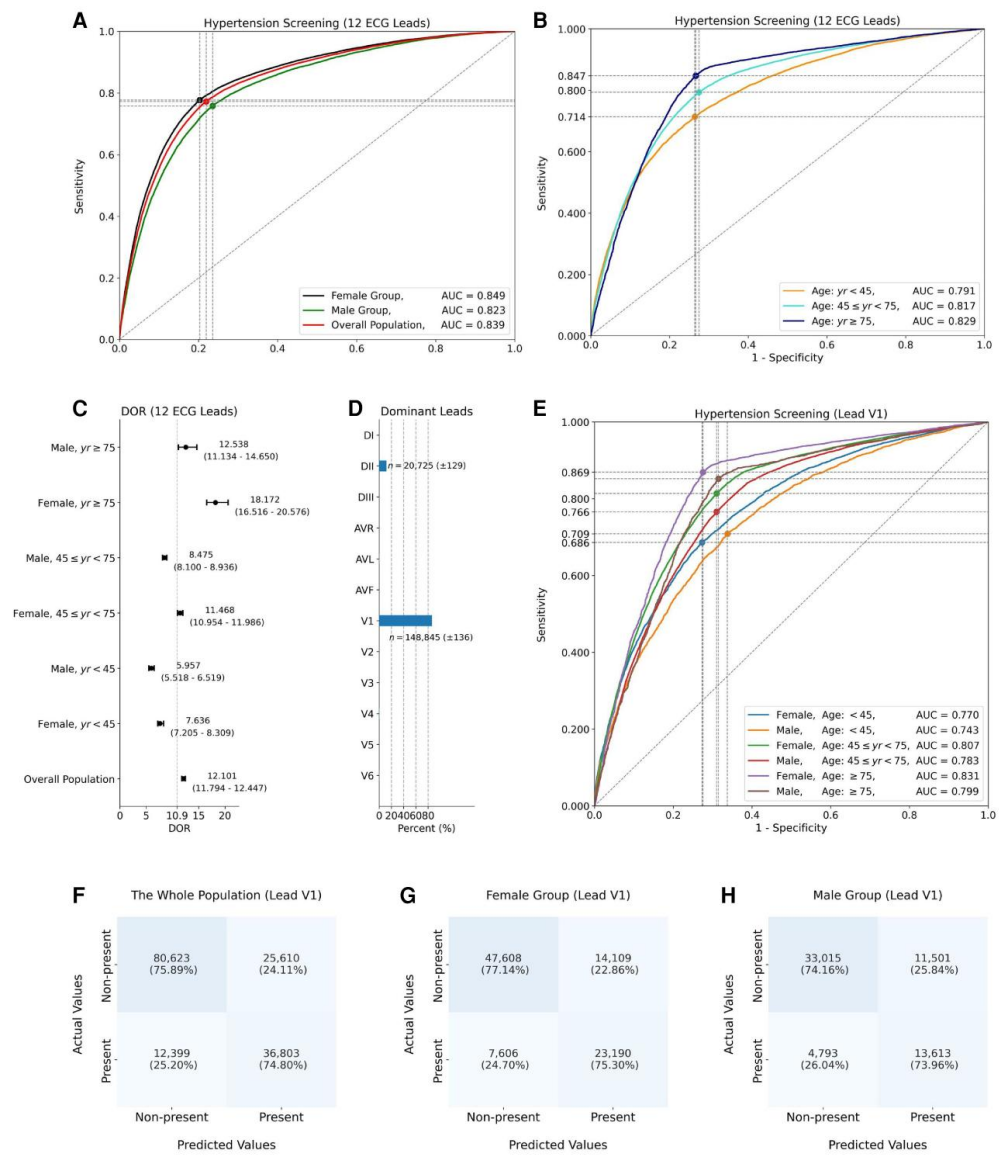
Model performance and lead importance for hypertension screening using our developed DNN model. (*A*) Performance comparison of the DNN model for hypertension screening using 12-lead electrocardiograms in terms of gender differences. (*B*) Performance comparison in terms of age differences using 12-lead electrocardiograms. (*C*) Diagnostic odds ratios with 95% confidence interval for hypertension screening in different populations. (*D*) Distributions of the dominant electrocardiogram leads (mean *±* standard deviation). (*E*) Performance comparison of hypertension screening using the dominant V1 lead. We demonstrate confusion matrices for hypertension screening using the dominant V1 lead in different population groups, including (*F*) the whole population, (*G*) the female group, and (*H*) the male group. The confidence interval and standard deviation are calculated by the bootstrap method.

In parallel with the previous two tasks, we implement the third task of hypertension screening using our developed DNN model. It can be seen from *Figure 5A* that the DNN model achieves an AUC score of 0.839 (95% CI, 0.837–0.841) and a DOR of 12.101 (95% CI, 11.794–12.447) in screening subjects with hypertension. Considering the effects of age and gender on the development of hypertension,^26^ we investigate the model performance of screening hypertension in different populations. It is shown in *Figure 5A* that the model achieves an AUC score of 0.849 (95% CI, 0.847–0.852) for hypertension screening in the female group, which is slightly higher than the AUC score of 0.823 (95% CI, 0.820–0.827) in the male group (*P* = 0.011). In terms of age differences, as shown in *Figure 5B* and *C*, the model has the highest performance in the old-age female group (*P <* 0.01), with an AUC score of 0.829 (95% CI, 0.822–0.836) and the DOR of 18.172 (95% CI, 16.516–20.576).

We identify dominant ECG leads for hypertension screening using the salient features produced by our DNN model. It can be seen from *Figure 5D* that our DNN model identifies the DII and V1 leads as the dominant ECG leads. When using V1 lead alone, the DNN model obtains an AUC score of 0.816 (95% CI, 0.814–0.818) for hypertension screening, which is a similar result to the model performance of 0.839 (95% CI, 0.837–0.841) using 12 ECG leads. As shown in the confusion matrix in *Figure 5F–H*, the DNN model has an accuracy of 74.80% on screening hypertension in the whole population using V1 lead alone, and it has a higher accuracy of 75.30% in the female group than that of 73.96% in the male group. In a further step, we use two ECG leads to screen hypertension by including the additional DII lead, which achieves the highest AUC score of 0.835 (95% CI, 0.827–0.844) in the old-age female group (Supplementary material online, *Figure S18*). We present the detailed results of comparison in Supplementary material online, *Figures S20–S22* and *Tables S11–S13*, which include hypertension screening using 12 ECG leads and dominant ECG leads considering age and gender differences. For the male group, we show that the DNN model obtains AUC scores of 0.802 (95% CI, 0.798–0.806), 0.812 (95% CI, 0.808–0.816), and 0.823 (95% CI, 0.820–0.827) using the V1 lead, DII and VI leads, and the 12 ECG leads separately. For the female group, we obtain AUC scores of 0.826 (95% CI, 0.823–0.829), 0.837 (95% CI, 0.834–0.840), and 0.849 (95% CI, 0.847–0.852) by using the three types of ECG leads. The results in Supplementary material online, *Figures S20–S22* and *Tables S11–S13* demonstrate the effectiveness of our identified dominant ECG leads for hypertension screening.

### Mortality risk stratification

**Figure 6:**
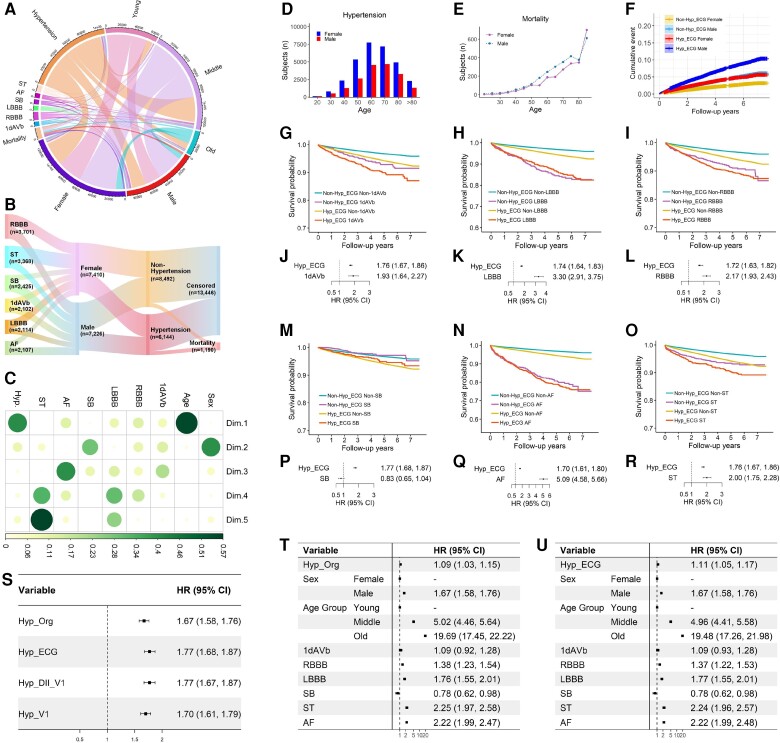
Mortality risk stratification for cohorts with age and gender differences, cardiac abnormalities, and hypertension. Panel (*A*) illustrates the prevalence and relationship of cohorts with the different types of covariates. Panel (*B*) shows the relationship between cardiac abnormalities and mortality considering the impact of gender differences and hypertension. Panel (*C*) ranks feature importance using the principal component analysis method. The principal component analysis features were calculated from the original electronic health records, showing the relative importance and complex relationships between demographic features, cardiac abnormalities, gender differences, and hypertension. Panels (*D*) and (*E*) demonstrate gender differences in cohorts with hypertension and mortality records. Panel (*F*) shows the cumulative event with increasing follow-up years. Panels (*G*–*I*) and (*M*–*O*) show the Kaplan–Meier survival curves for different population cohorts. Panels (*J*–*L*) and (*P*–*R*) are hazard ratios adjusted for hypertension and cardiac abnormalities. Panel (*S*) shows univariate hazard ratios for cohorts with hypertension in the original data set (Hyp_Org) and our electrocardiogram-predicted results, using 12 leads (Hyp_ECG), 2 leads (Hyp_DII_V1), and 1 lead (Hyp_V1). (*T*) and (*U*) are hazard ratios adjusted for age, gender, cardiac abnormalities, and hypertension. HR, hazard ratio; Hyp_Org, hypertension records in the original data set; Hyp_ECG, electrocardiogram-predicted hypertension using 12 leads; Hyp_DII_V1, electrocardiogram-predicted hypertension using DII and V1 leads; Hyp_V1, electrocardiogram-predicted hypertension using V1 lead.

In a further step, we implement the fourth task of stratifying the risk of mortality using our ECG-predicted results. We show the impact of cardiac abnormalities, gender differences, and hypertension on the risk of mortality in *Figure 6*. In particular, as shown in *Figure 6D* and *E*, we demonstrate gender differences in the development of hypertension and mortality, where middle-aged males have a higher rate of mortality than middle-aged females, and the hypertensive male group has the highest mortality rate among all the population groups.

We compare the risk of mortality for hypertensive subjects estimated using the original data set and our ECG-predicted results. As shown in *Figure 6S*, the cohort is estimated with the mortality risk of a univariate hazard ratio (HR) of 1.77 (95% CI, 1.68–1.87) using our ECG-predicted results, which is similar to the HR of 1.67 (95% CI, 1.58–1.76) for the hypertensive cohort in the original data set, indicating the effectiveness of our ECG-predicted results for the mortality risk stratification. In particular, we show that using V1 lead alone, or the DII and V1 leads, our ECG-predicted results obtain similar risk ratios to that of the cohort in the original data set, indicating that the two ECG leads can be used efficiently for mortality risk stratification for the hypertensive cohort. Meanwhile, we investigate the risk of mortality considering all covariates, such as cardiac abnormalities, age, gender, and hypertension. *Figure 6T* shows the adjusted HRs for the risk of mortality for the cohort in the original data set, and *Figure 6U* shows the adjusted HRs for the mortality risk using our ECG-predicted results. The comparable values between the adjusted HRs in *Figure 6T* and *U* further indicate the effectiveness of our ECG-predicted results for mortality risk stratification.

In addition, we investigate the risks of mortality for different population groups using our ECG-predicted results, facilitating personalized healthcare of risk management. As shown in *Figure 6K* and *Q*, we identify the highest mortality risk for the cohort with AF (adjusted HR = 5.09, 95% CI, 4.68–5.66), followed by cohort with LBBB (adjusted HR = 3.30, 95% CI, 2.91–3.75). However, as indicated in *Figure 6H* and *N*, the survival probabilities for the cohorts with AF and LBBB are less affected by hypertension. For the population with 1dAVb and ST, as shown in *Figure 6J* and *R*, the two cohorts have high risks of mortality with adjusted HRs of 1.93 (95% CI, 1.64–2.27), and 2.00 (95% CI, 1.75–2.28) separately. Nevertheless, as shown in *Figure 6G* and *O*, the survival probabilities for the two cohorts are affected by hypertension; In particular, the subjects have higher risks of mortality when they have hypertension concurrently. Using ECG-predicted results, we also investigate the risks of mortality for different population groups considering cardiac abnormalities, gender differences, and hypertension, in Supplementary material online, *Figures S23–S25*. As indicated in Supplementary material online, *Figure S23C*, males have a higher risk of mortality than females for the cohorts with LBBB; and as shown in Supplementary material online, *Figure S25C*, both males and females have a high risk of mortality for the cohorts with AF.

## Discussion

In this study, we developed an ‘end-to-end’ DNN model with state-of-the-art performance on cardiac diagnosis using a large data set with 2.3 million ECG recordings, the ECG-predicted results are then used for mortality risk stratification. There are studies in the literature using AI models for ECG interpretation.^13,14,28,29^ For instance, the study by Raghunath *et al*.^13^ developed a DNN model to predict all-cause mortality, and provided interpretation of ECG morphological features for the prediction. However, the research has several limitations on interpreting the ECGs. First, the study used multiple leads as a combination for model input, and the learned saliency map was shared by several leads instead of the weight for each specific ECG lead. Although previous study used a guided-back propagation technique to derive lead-wise weight,^30^ the generated heatmap for the ECG recording was discrete, e.g. highlighting adjacent data in the ECG morphology as disconnected points in the heatmap. As a result, it is difficult to interpret the visualized ECG features produced by the DNN model. Second, it is understood that ECG morphology may change over time. As demonstrated in *Figure 3B* in our study, some morphologies (e.g. P waves) are not constantly presented in the ECG recording. Thus, the interpretation of ECGs needs to be flexible and accurate over time. Moreover, the study by Raghunath *et al*.^13^ demonstrated the interpretation of ECG data segments with a short duration (i.e. 0.6 s), and it is unclear how the interpretability changes over time when standard or longer duration ECG recordings are used. In this research, we proposed a new *isolation-integration* strategy for ECG interpretation, enabling to learn feature importance for each ECG lead precisely rather than the shared weights as presented in Raghunath *et al*.^13^ More importantly, as presented in Section 3.1, we show the flexibility and robustness of our developed DNN model for identifying changes in ECG features over time in a variety of diagnostic tasks.

We used a stepwise strategy to validate the effectiveness of our developed DNN model for ECG interpretation, progressing from well-established tasks to rare medical diagnoses. (i) We first implemented the task of interpretable diagnosis of cardiac arrhythmias, where the existing textbook knowledge can be used to validate our findings. For example, the V1 lead was observed having dominant waves for the diagnosis of ventricular arrhythmias,^31^ and lateral leads, such as the V5 lead, were considered to be important in the diagnosis of bundle branch blocks.^32^ Consistent with prior knowledge, our findings also provide new insights into the diagnosis of arrhythmias. For instance, our DNN model highlights the importance of U waves in identifying the SB. Notably, prominent U waves were also reported in asymptomatic SB in the literature,^33^ whereas the U waves are difficult to observe due to their low amplitudes, which highlights the advantage of our model in computerized ECG interpretation. (ii) After validating our developed interpretable model for the diagnosis of ECG abnormalities, we extended the study in a wider context, i.e. the second task of gender classification, and the third task of hypertension screening. Again, the previous study indicated the importance of V5 lead for gender identification,^29^ validating the findings of our identified dominant leads. Furthermore, we note that previous studies showed that high blood pressure is in association with an increased risk of AF,^34^ which could potentially explain the AF and hypertension having similar dominant ECG leads in our study. In particular, it was observed that the reduction of R-wave amplitude in V1 lead by *≥* 1 mm after three months of treatment was related to better survival.^35^ Previous studies also indicated that P wave in V1 lead and Cornell product were associated with diastolic blood pressure.^36^ These studies could explain our findings that the V1 ECG lead plays a crucial role in hypertension screening and the stratification of mortality risk in hypertensive cohorts.

We note that previous studies used ECGs to predict mortality and identified ST-wave elevation, T-wave morphological features, and QRS micro-fragmentation as risk indicators.^13,37^ However, mortality risk stratification in patients with cardiovascular diseases requires considering numerous factors such as age, gender differences, health conditions, and comorbidities. In this study, we implemented Tasks #1–#3 using the ECG data to validate the diagnostic performance and interpretability of our developed DNN model. In a further step, as a proof-of-concept study, we additionally performed Task #4 to demonstrate how to use our ECG-predicted results for healthcare applications, such as the stratification of mortality risk. We believe that the DNN model developed in this study will contribute to a more comprehensive evaluation of mortality risks by taking into account these diverse factors alongside the cardiac diagnosis and interpretation. In addition, our research provided new insights for the stratification of mortality risk using reduced-ECG leads. For example, it could be potentially used to develop more convenient devices for monitoring mortality risk rather than collecting the standard 12-lead ECGs for the prediction.

Recent advances in ECG technologies have enabled the development of small, low-cost, and easy-to-use wearable devices, which typically use a subset of the standard 12 leads for remote monitoring. However, as highlighted in the PhysioNet/Computing in Cardiology Challenge,^15^ there is limited research to demonstrate that reduced-lead ECGs can capture the wide range of diagnostic information achieved by the 12-lead ECGs. This study provides substantial evidence to show that our developed DNN model enables to automatically identify important ECG leads for a variety of medical tasks. By implementing extensive comparison studies between our identified dominant leads and the 12 ECG leads, we show that our identified reduced-lead ECGs can achieve comparable performance with the standard 12-lead ECGs for lead-specific and disease-specific diagnosis, which can meaningfully contribute to the development of reduced-lead wearable devices for cardiac monitoring. In addition, our proposed DNN model demonstrates the effectiveness of hypertension screening using reduced-lead ECGs, indicating the potential application of cuffless blood pressure monitoring as a future direction.

Our work is perhaps best understood in the context of its limitations. (i) We note that there is a broad range of heart arrhythmias, and a limited category of abnormalities was tested in this study, as they are considered to be representative of both rhythmic and morphological abnormalities. Notably, the computerized interpretation facilitated by our developed DNN model does not depend on any prior assumptions regarding a specific category of cardiac abnormalities. Consequently, our developed for ECG interpretation could potentially be applied to other abnormalities when they become accessible in the future. (ii) We recognize that it is difficult to accurately label the data set with such a sheer scale, leading to the impact on model performance evaluation. The limitation could be mitigated by evaluating the model across multiple data sets or using unsupervised learning for label refinement in our future study. (iii) We used ECG-predicted results for mortality risk stratification. Nevertheless, previous study showcased the use of raw ECGs as an outcome predictor,^38^ providing insights for us to advance our model by integrating a loss function for survival analysis. (iv) Apart from hypertension screening as presented in this study, there are also other critical health conditions such as heart failure and coronary disease that are associated with the risk of mortality; Meanwhile, the prediction of health conditions could be potentially affected by confounding factors, such as race disparities.^39^ Our future research will use the developed model for more comprehensive healthcare evaluation on a broader scale.

## Conclusion

This study developed an end-to-end deep-learning model with state-of-the-art granularity for interpretable cardiac diagnosis using ECG recordings, and the results predicted by the ECGs were then used for mortality risk stratification. By leveraging a large and diverse data set consisting of 2.3 million ECGs collected from 1.6 million subjects with 7 years follow-up, we validated the model performance across a range of medical tasks, including arrhythmia diagnosis, gender identification, hypertension screening, and mortality risk stratification. Our model shows substantial advantages to promote explainable diagnosis with cardiologist-level performance outperforming the benchmark model, and the advance in mortality risk stratification. Furthermore, the model demonstrates the potential to discover novel patient-relevant information from clinical data measurements.

## Supplementary Material

ztae014_Supplementary_Data

## Data Availability

As the original data set of ECG recordings is prohibitively large for upload, about 15% of the CODE data set has been made openly available as annotations (https://doi.org/10.5281/zenodo.4916206), including 345 779 ECG recordings collected from 233 770 subjects. The testing data set and the labels are also publicly accessible (https://zenodo.org/record/3765780#.YbIaypHP 36c). The external testing data set is available from the PhysioNet/CinC 2017 Challenge (https://archive.physionet.org/physioba nk/database/challenge/2017/).
