# Supplementary material for: Decoding 2.3 million ECGs: interpretable deep learning for advancing cardiovascular diagnosis and mortality risk stratification
Source: Eur Heart J Digit Health. 2024 Feb 19;5(3):247–59. doi: 10.1093/ehjdh/ztae014 (PMC11104458; doi:10.1093/ehjdh/ztae014)
Supplement: ztae014_Supplementary_Data [file ztae014_supplementary_data.pdf]

Supplementary Material

Title: Decoding 2.3 Million ECGs: Interpretable Deep Learning for Advancing Cardiovascular Diagnosis and Mortality Risk Stratification

A1. Data Acquisition and Annotation

The present study uses a dataset consisting of standard 12-lead ECG recordings collected by the Telehealth Network of Minas Gerais (TNMG), a public healthcare system to provide tele-consultation and tele-diagnosis for 811 municipalities in the state of Minas Gerais, Brazil [1, 2]. The ECG recordings were mostly collected in primary care facilities during clinic visits with follow-ups from 2010 to 2016, which were performed either using the tele-electrocardiogram machine of model TEB (Tecnologia Eletronica Brasileira, Sao Paulo, Brazil), or the ErgoPC 13 (Micromed Biotecnologia, Brasilia, Brazil). This study complies with all relevant ethical regulations, and the Research Ethics Committee of the Universidade Federal de Minas Gerais (Protocol 49368496317.7.0000.5149) gave the ethical approval.

The ECG tests were recorded for a duration of 7 to 10 seconds with sampling frequencies ranging from 300 to 600 Hz. To ensure consistency of the data format, the recordings were resampled with 400 Hz, and then zero-padded to the length of 4,096 data points. The rescaled ECG recordings were stored in a structured database, namely the Clinical Outcomes in Digital Electrocardiology (CODE). A dataset of 2,322,513 ECG recordings was retrieved from the CODE database. We excluded low-quality ECGs (n_{ECGs} = 6,731) that had zero values for more than 80% of the data points, and used a total of 2,315,782 ECG recordings for the current study.

We obtained electronic health records for subjects in the CODE dataset by performing a link matching between the ECG tests and the national public databases of the Mortality and Hospitalization Information Systems, using a standard probabilistic linkage method (FRIL: Fine-grained record integration and linkage software, v.2.1.5, Atlanta, GA) [1, 3]. Arterial hypertension in the data records was defined as a systolic blood pressure $\geq$ 140 mm Hg, or diastolic blood pressure $\geq$ 90 mm Hg, or self-declared use of anti-hypertensive medication. The data were anonymised after the linkage matching.

Annotation of ECG recordings in the CODE dataset was performed by both trained professionals and computerised software using the following procedures, (*i*) the sampled ECG recordings were first sent by internet to central servers, and a team of trained professionals used standardised criteria to generate free-text ECG reports, which were digitally recognised by a hierarchical free-text machine learning method [2]. The ECG reports were periodically audited by professionals to recognise medical errors and discordant interpretations; (*ii*) The Glasgow 12-lead ECG analysis program was used to analyse the ECG recordings, and generate the diagnosis results of the Glasgow Diagnostic Statements and Minnesota Code; (*iii*) The presence of a specific ECG abnormality was automatically considered when there was an agreement between the cardiologist report and the computerised diagnosis result. A manual review was performed when the two sources of diagnosis disagreed [1].

The holdout unseen testing dataset for model evaluation was independently reviewed by two certified cardiologists, and the data label was obtained when annotations from the two professionals were matched; Where annotations did not match, a specialist was introduced to decide the diagnosis. We present the evaluation results of the two senior professionals in the main text of this paper. We also calculate the Cohen's kappa coefficient of the evaluation results from the two senior professionals [2]; values are 0.741 for 1dAVb, 0.955 for RBBB, 0.964 for LBBB, 0.844 for SB, 0.831 for AF, and 0.902 for ST. These values demonstrate the inter-rater agreement for the two professionals, and we therefore use these evaluation results as the data labels. The testing dataset was also reviewed by three groups of junior professionals, i.e., two 4th year cardiology residents, two 3rd year emergency residents, and two 5th year medical students. To reduce the bias of ECG evaluation, the two professionals in each of the three groups were asked to annotate half of the testing dataset, and the concatenated performance scores were obtained for the three

groups of junior professionals. We acknowledge the inherent difficulty in annotating ECG records, with observed inter-observer variations even among certified cardiologists. These variations can impact the model evaluation. In this study, we obtained the reference standard by involving three cardiologists for the data labelling. Future evaluation will implement continuous quality check, expert panel reviewing, and refining annotations, which will mitigate individual biases and uphold the high standard of reference labels.

A2. Model Development and Statistical Analysis

Developing Interpretable DNN Model with Fine Granularity. The architecture of our DNN model is illustrated in Extended Figures S1, and we developed a refined gradient-weighted class activation mapping (Grad-CAM) module for ECG interpretation with a fine granularity. The Grad-CAM model has been widely used for image interpretation [4], however, as demonstrated in previous research, the target objects detected by the Grad-CAM model include many irrelevant features [5]. This is mostly due to the following reasons: (*i*) *Dimension alignment*. Generally, a deep learning model uses pooling layers to reduce the dimension of input data, as a result, the heatmap calculated from the last Conv layer has a smaller size than the input data. To match the dimension between the learned heatmap and the input data, the linear mapping must be used for the alignment. (*ii*) *Weight sharing*. Other than the dimension alignment of the heatmap, weight sharing across different ECG leads in a deep learning model also affects the interpretive ability. For instance, the Conv kernels of the DNN model in the previous research learned kernel weights across all ECG leads, rather than the weights for a specific lead.

We note that previous studies indicated the significance of individual or a subset of ECG leads for cardiac diagnosis. For instance, the DII lead holds diagnostic importance for AF [6], the V1 lead exhibits predominant waves in the diagnosis of ventricular arrhythmias [7], and the lateral leads are deemed crucial in diagnosing bundle branch blocks [8]. These findings suggest that specific subsets of ECG leads can play pivotal roles in cardiac diagnosis. This insight motivates us to develop a DNN model capable of independently learning features from individual ECG leads. Moreover, when employing a composite input of 12 ECG leads for the model, the computed gradients are shared across all leads. Consequently, it is difficult to interpret each lead precisely using the shared kernel weights.

With the above considerations, we develop the following techniques to obtain a fine resolution for the ECG interpretation. In particular, we propose an *isolation-integration* strategy to allow the deep learning model to learn lead-wise weights for the ECG recording. The strategy is defined in the following steps:

(*i*) During the isolation stage, in order to reduce the effect of weight sharing on the interpretation, we proposed to separate each of the 12 leads in an ECG recording, and then use each isolated lead as an independent input to the model. This strategy allows the DNN model to learn features precisely for each separated ECG lead, rather than shared weights across multiple channels;

(*ii*) During the integration stage, we develop a stepwise strategy to combine the learned features from each ECG lead, which enables the DNN model to explore elaborate relationships between different ECG leads, and prompts a comprehensive decision for diagnosis using the combined information. Some previous research used a global pooling layer for feature integration, however, the temporal information of the learned features would be lost due to this global pooling [9];

(*iii*) For the dimension alignment, it is important to ensure a similar size between the calculated heatmap and the input data, which will reduce the effect of data alignment on visualising salient features. As the dimension reduction of a DNN model is mostly from the pooling process, we therefore use a minimum number of pooling layers in the feature learning stage. This results in the kernel size of the last Conv layer having a close dimension to the input data, and therefore produces a refined resolution for the interpretation.

Model Development and Training. Following our developed *isolation-integration* strategy, we first separate each of the 12 leads in the ECG recording, and use the isolated leads as inputs to the deep learning model; Then, we develop modules using residual neural networks to learn latent features for each ECG lead, and use the bi-direction long short-term memory model (BiLSTM) to learn temporal information, as well as the relationships between different ECG leads, and the integrated features of the 12 ECG leads are used for the model prediction. As illustrated in Extended Figure S1, our developed DNN model has 12 channels for the model inputs, with each channel corresponding to one ECG lead. For each isolated input channel, we first use a Conv layer with 16 kernels to learn latent features from raw data of the ECG lead, which is followed by a batch normalisation (BN) layer, a rectified linear unit (ReLU) activation layer, and a max pooling layer. Next, we use four residual blocks to learn deep features from each lead, and each of the residual blocks consists of four repeated modules with the BN, ReLU, and Conv layers. Particularly, in the first two residual blocks, the Conv layer has 16 kernels with a width size of 16; In the remaining two residual blocks, the Conv layer has 48 kernels with a width size of 48. After the second residual block, we use a Conv layer with 48 kernels to align dimensions with the following third residual block. At the end of each channel, we use a Conv layer with 48 kernels to finish feature learning for the ECG lead.

After learning features from the isolated input channels, the features are processed in the integration stage as illustrated in Extended Figure S1. We stepwise integrate the features to learn elaborate relationships between different ECG leads. We generate a large feature matrix by concatenating the learned features from each of the isolated channels. As there is only one pooling layer used for each input channel in the isolation stage, the temporal dimension of the generated feature matrix in the concatenate layer is half the size of the input ECG recording, which enables us to learn detailed weights for the ECG morphologies. We note that the last Conv layer in each channel has the size of 48 kernels, and the generated feature matrix has a dimension of 576, which is obtained by concatenating Conv layers in the 12 ECG leads. Next, we learn relationships between different ECG leads using the BiLSTM block and two time-distributed dense layer (TD Dense) blocks. The TD Dense blocks consist of a max pooling layer (MaxP), an average pooling layer (AvgP), and a dropout layer. The BiLSTM block consists of two layers, one has a forward direction and the other has a reverse direction, and each of the two layers has 64 cells in the hidden state. For the two TD Dense blocks, the first one has 64 units and the second block has 32 units. We then flatten layers of the TD Dense block followed by a fully connected layer with 128 units. Finally, we use a sigmoid function to calculate the probability for the output of model prediction. The developed DNN model is used to perform the diagnostic tasks in this study, i.e., ECG abnormality diagnosis, gender identification, and hypertension screening. We train the model independently for each of the diagnostic tasks, whilst keeping the model architecture and hyperparameters the same for all the tasks, i.e., the number of neurons, activation function, optimizer, batch size, and epochs. For the first task, the DNN model has an output vector of six values, indicating the six types of ECG abnormalities; For the second task, the model has an output of a single value, indicating the probability of male or female; For the third task, the model also has an output of a single value, indicating the probability of hypertension presented for the

subject.

Hyperparameter Tuning. The neural network was trained using the loss of binary cross-entropy, which was minimized by the Adam optimizer with default parameters [10]. Hyper-parameters of the network architecture were chosen via a combination of grid search and manual tuning with the following considerations, the number of residual blocks $\{2, 3, 4\}$, kernel size for the Conv layer $\{8, 16, 32, 48\}$, the size of pooling layers $\{2, 4\}$, the number of Conv layers $\{1, 2, 4\}$ in each residual block, dropout rate of $\{0, 0.2, 0.5, 0.6\}$, the mini batch size of $\{32, 64, 128\}$, the initial learning rate of $\{10^{-2}, 10^{-3}, 10^{-4}\}$, the number of epochs without improvement in plateaus between 7 and 15, which would result in a reduction of the learning rate by a factor of 10. After tuning the parameters with a stratified subset of 300K samples from the original dataset, we set a learning rate of 10^{-4} and use the whole dataset to train the model with a mini batch size of 128 samples, and the maximum number of epochs was set as 70. During the model training, a holdout set with 10% of the data was used for the validation. We tried different configurations of the model development, especially in the feature integration stage, such as the BiLSTM, LSTM, and TD Dense layers; and found that the combination of BiLSTM with two TD Dense layers shows good performance for the diagnosis. To reduce the effect of imbalanced classes in the dataset, we weighted each sample by multiplying a score of 2*log(n_{ECGs} / n_{Class}), where n_{ECGs} indicates the total number of samples, and n_{Class} is the size of samples in the class. A total of 20 Nvidia V100 GPUs in a high-performance computing platform are available to train the DNN model, which is located at the Institute of Biomedical Engineering, Department of Engineering Science, University of Oxford.

Survival Model. We use the Cox proportional regression model for the mortality analysis [11], and report hazard ratios (HR) with 95% confidence intervals (CI) for the risk of mortality. We perform univariate and multivariate analyses considering covariates including cardiac abnormalities, age, gender, and hypertension. As hypertension is the largest single contributor to CVDs [12], we investigate and present the Kaplan-Meier survival curves for the hypertension group, and calculate the HRs for hypertensive cohorts adjusted by gender and cardiac abnormalities. All of the survival analyses were performed using the R statistical platform version 4.2.2.

Statistical Analysis and Model Performance. To evaluate the performance of our developed DNN model on the medical tasks, we calculate standard matrices of the testing results for each independent task. We compute the area under the receiver operating characteristic curve (AUC-ROC) to report the model performance; we also calculate the F1-score for the first task, as it has an imbalanced testing dataset, and the score is used to compare the performance of our model with the evaluation results from the cardiology professionals and the state-of-the-art model [2]. We calculate the micro average across different classes to report an overall score of the model performance, which computes the total true positives, false negatives, and false positives to obtain a comprehensive metric. The optimal cut-off point for the sensitivity and specificity scores is obtained by maximising the G-mean value, which is a geometric mean of the two scores [13]. We use the diagnostic odds ratio (DOR) to indicate the model's ability of diagnosis, which is calculated as the positive likelihood ratio (sensitivity / (1-specificity)) to the negative likelihood ratio ((1-sensitivity) / specificity). A value of DOR larger than 1 indicates the model having a discriminatory test performance, with the DOR value correlating positively with better diagnosis performance [14]. We use the bootstrap method (repeated sampling 1,000 times) to compute the 95% CI and standard deviation for the calculated indices [5]. As suggested by the previous research on comparing evaluation results for cardiac diagnosis [2], we report two-sided McNemar's χ^2 test to evaluate differences between classification results for paired samples, and use Pearson's χ^2 test to evaluate differences for unpaired samples. We also calculate

Cohen's kappa coefficient to test inter-rater/-model agreement [15]. We consider a p-value of less than 0.05 as statistically significant. We note that we also observed significant differences between Kaplan-Meier curves using the log-rank test, and the significance of HRs using the Cox regression model for the mortality risk analysis [11]. However, some recent research showed that it is arguable to report p-values in medical research and practice [16]. Therefore, we mostly present the 95% CI and variance of our results to show the statistics.

A3. Extended Tables and Figures

Extended Table S1: Dataset and study population for the three diagnostic tasks in this study (Numbers and percentages).

			Training (90%) + Validation (10%)	Testing
**Task #1**	Numbers of ECGs (Task #1):		n = 2,315,728	n = 827
	Abnormality	1dAVb	35,755 (1.54%)	28 (3.39%)
		RBBB	63,522 (2.74%)	34 (4.11%)
		LBBB	37,166 (1.60%)	30 (3.63%)
		SB	37,904 (1.64%)	16 (1.93%)
		AF	41,776 (1.80%)	13 (1.57%)
		ST	49,852 (2.15%)	37 (4.47%)
	Age	$yr < 45$	706,764 (30.52%)	225 (27.21%)
		$45 \leq yr < 75$	1,321,650 (57.07%)	500 (60.46%)
		$yr \geq 75$	287,368 (12.41%)	102 (12.33%)
	Gender	Male	920,321 (39.74%)	321 (38.81%)
		Female	1,395,461 (60.26%)	506 (61.19%)
**Task #2**	Numbers of ECGs (Task #2):		n = 1,398,907	n = 155,435
	Age	$yr < 45$	488,946 (34.95%)	54,341 (34.96%)
		$45 \leq yr < 75$	762,286 (54.49%)	84,640 (54.45%)
		$yr \geq 75$	147,675 (10.56%)	16,454 (10.59%)
	Gender	Male	562,640 (40.22%)	62,922 (40.48%)
		Female	836,267 (59.78%)	92,513 (59.52%)
**Task #3**	Numbers of ECGs (Task #3):		n = 1,398,907	n = 155,435
	Hypertension	Present	442,918 (31.66%)	49,202 (31.65%)
		Non-present	955,989 (68.34%)	106,233 (68.35%)
	Age	$yr < 45$	488,946 (34.95%)	54,341 (34.96%)
		$45 \leq yr < 75$	762,286 (54.49%)	84,640 (54.45%)
		$yr \geq 75$	147,675 (10.56%)	16,454 (10.59%)
	Gender	Male	562,640 (40.22%)	62,922 (40.48%)
		Female	836,267 (59.78%)	92,513 (59.52%)

*The model for Task #1 was trained using the whole dataset; The models for Tasks #2 and #3 were trained using the dataset with unique patients, we randomly split the dataset into training and test with the ratio of 0.9: 0.1, and during the training process, we randomly select 10% of the training set for model validation.

Extended Table S2: Dataset and study population for the fourth task in this study (Numbers and percentages).

	Numbers of Subjects (Task #4): n = 155,435		
**Task #4**	Age	$yr < 45$	54,341 (34.96%)
		$45 \leq yr < 75$	84,640 (54.45%)
		$yr \geq 75$	16,454 (10.59%)
	Gender	Male	62,922 (40.48%)
		Female	92,513 (59.52%)
	Cohorts	Mortality	5,196 (3.34%)
		Censoring	150,239 (96.66%)

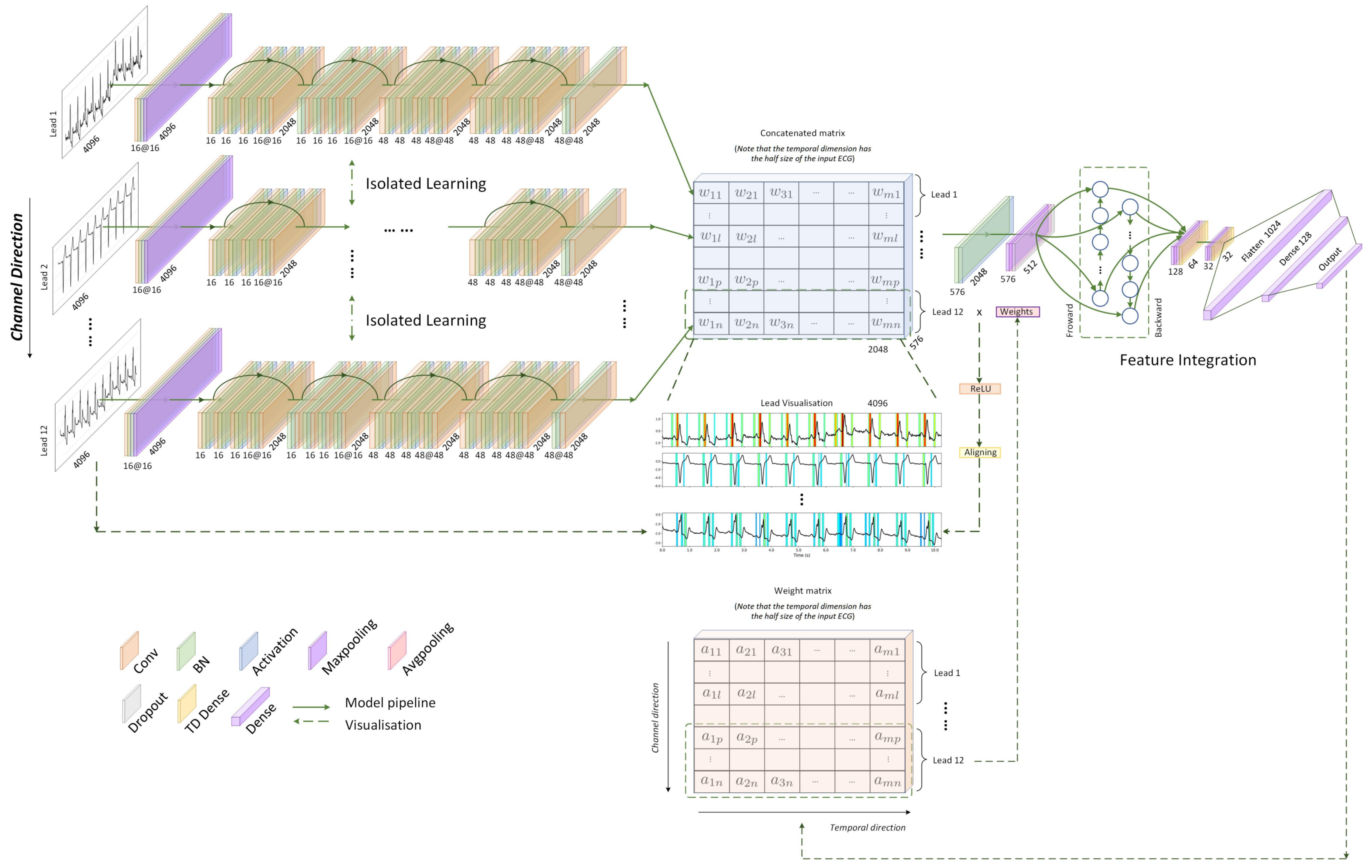

Extended Figure S1: Pipeline of our developed DNN model for the interpretable diagnosis using ECG recordings and salient feature visualisation with a fine resolution. We developed a new *isolation-integration* strategy for ECG interpretation, enabling to learn feature importance for each ECG lead precisely rather than shared weights. The designed concatenated feature matrix and weight matrix have a half length of the input ECG recording in the temporal direction, and this also contributes to producing salient features for fine-grained ECG interpretation.

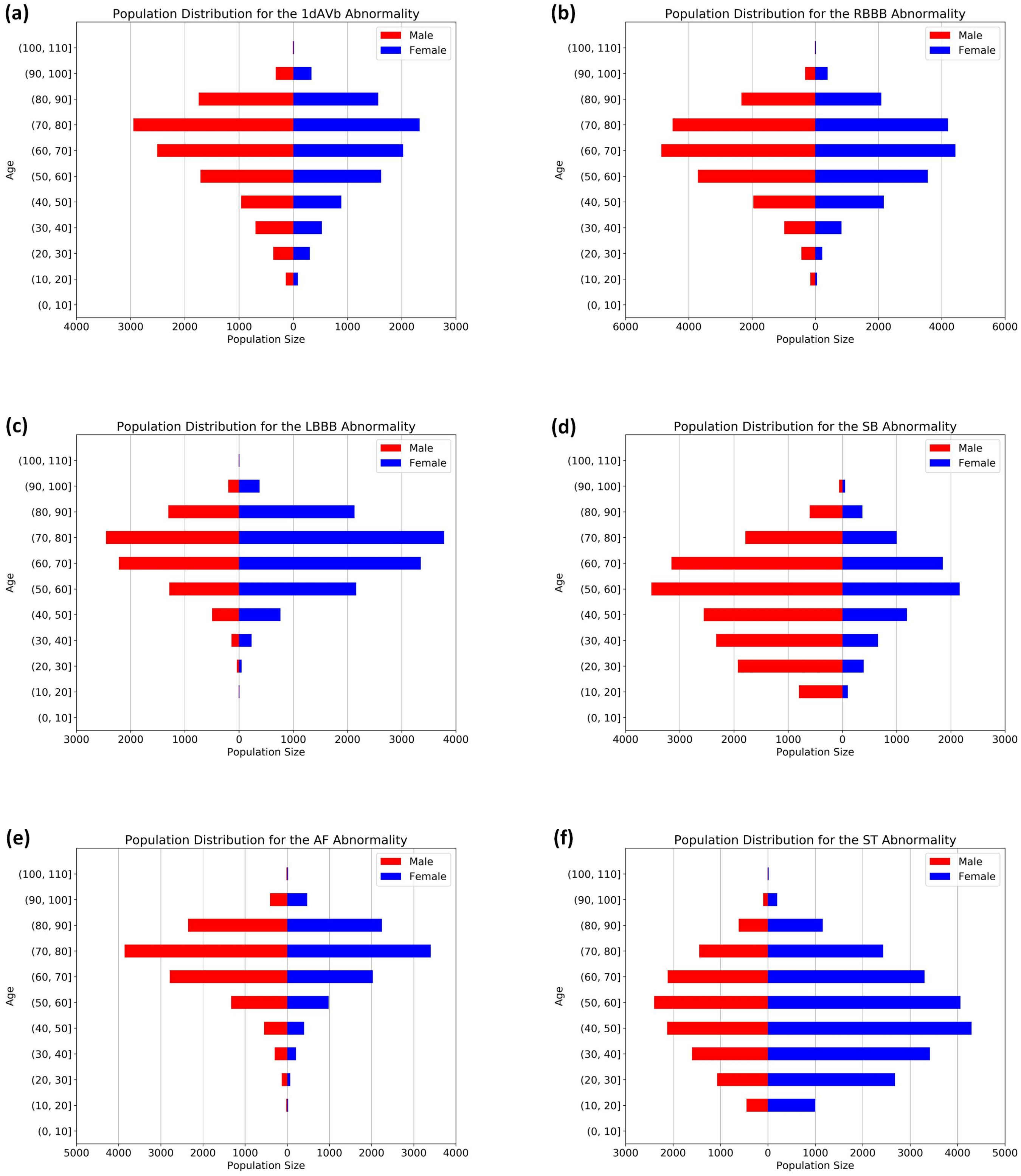

Extended Figure S2: Prevalence of ECG abnormalities in the CODE dataset (n_{Subjects} = 1,558,772), including **(a)** first-degree atrioventricular block (1dAVb), **(b)** right bundle branch block (RBBB), **(c)** left bundle branch block (LBBB), **(d)** sinus bradycardia (SB), **(e)** atrial fibrillation (AF), and **(f)** sinus tachycardia (ST).

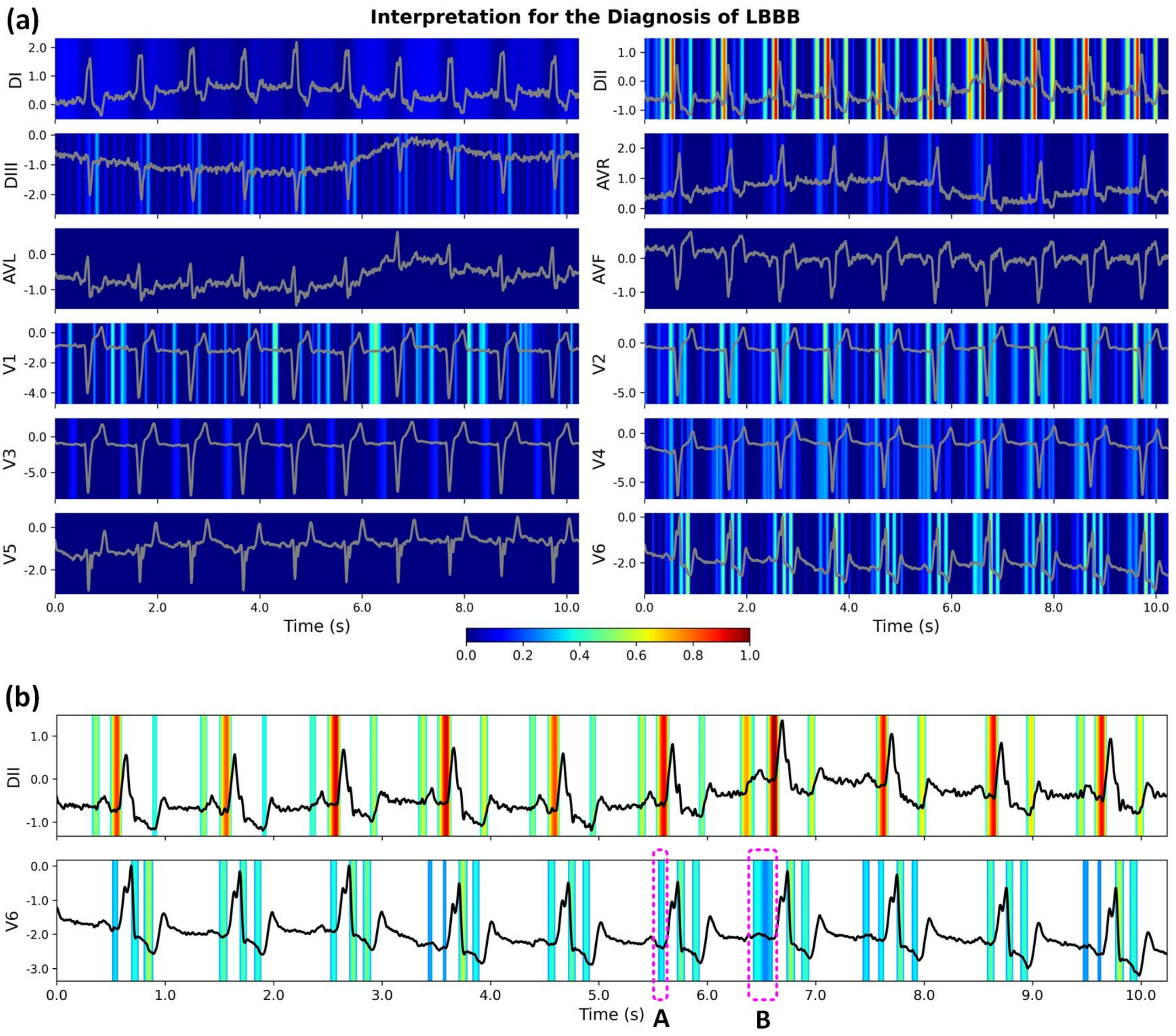

Extended Figure S3: Interpretation for the diagnosis of left bundle branch block (LBBB) using the proposed DNN model. **(a)** The original calculated heatmaps for 12 ECG leads, with colour bar ranging from blue to red indicating the increasing weights of importance. **(b)** The refined view of heatmaps for the DII and V6 leads with background colour removed. The diagnosis criteria of LBBB include the absence of Q waves in lateral leads, notched R waves in lateral leads, and T wave inversion [8]. Our developed DNN model recognises these pathological morphologies successfully in the V6 lead. The model also identifies other salient waves in the DII lead, such as the absence of Q waves, T inversion, and the segments before P waves. Using the combination of salient waves in the 12 ECG leads, the DNN model diagnoses the LBBB with the probability of 0.974; While with the removal of the DII lead, we obtained a prediction probability of 0.886, indicating additional knowledge derived from DII was missing. Notably, our model is flexible in identifying the pathological morphologies. For example, the morphologies in segments A and B have different time durations, and the DNN model identifies the varying lengths for the two segments successfully in the V6 lead.

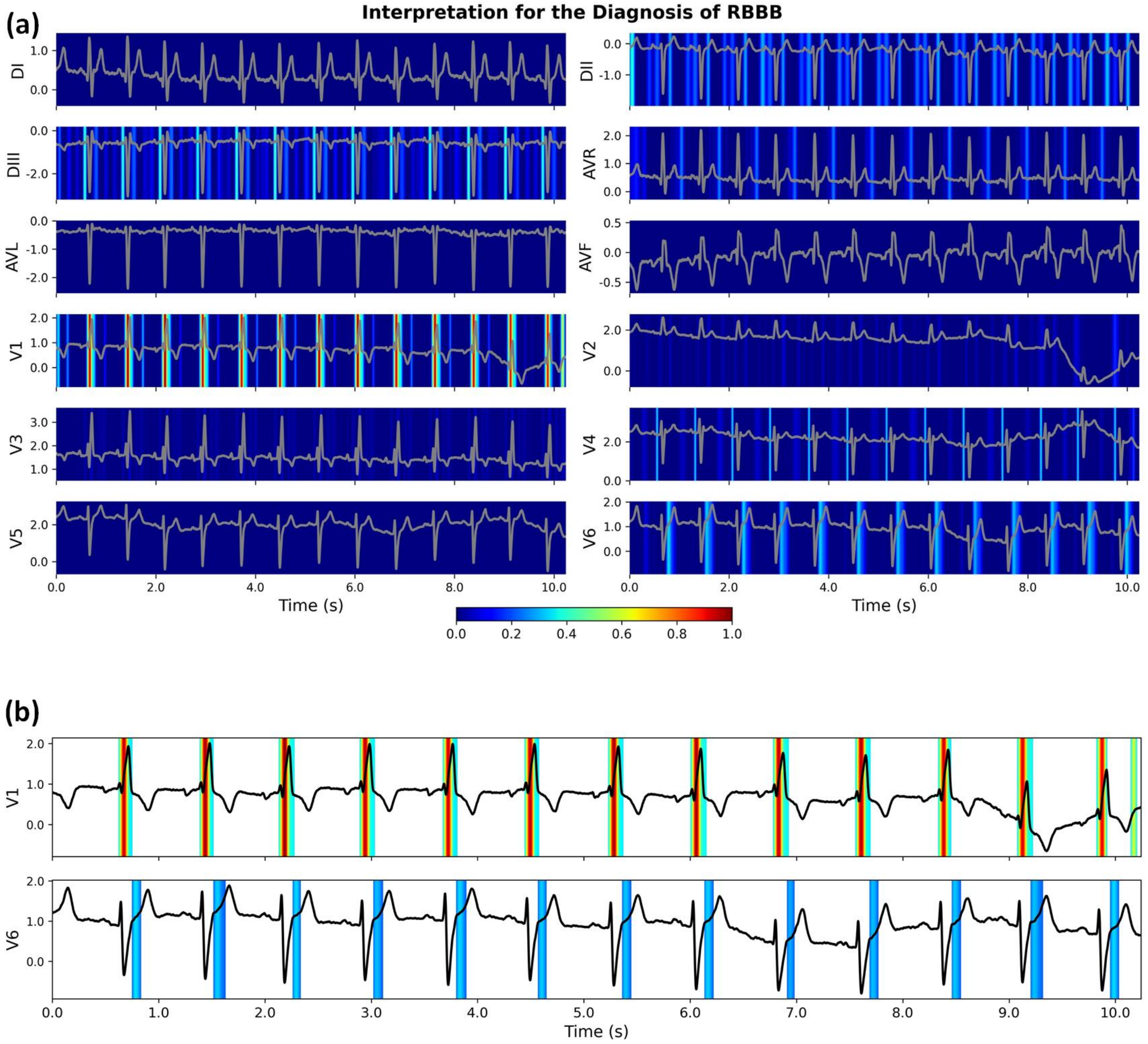

Extended Figure S4: Interpretation for the diagnosis of right bundle branch block (RBBB) using our DNN model. **(a)** The original calculated heatmaps for 12 ECG leads, with colour bar ranging from blue to red indicating the increasing weights of importance. **(b)** The refined view of heatmaps for V1 and V6 leads with background colour removed. For the diagnosis of RBBB, the RSR′ pattern in the anterior precordial leads is an important criterion [8]. Our developed DNN model recognises the ‘M-shaped’ QRS complexes successfully in the V1 lead, and it also highlights the importance of S waves in the V6 lead. Using the combined salient features, the DNN model has a probability of 0.929 to diagnose the RBBB with the ECG recording.

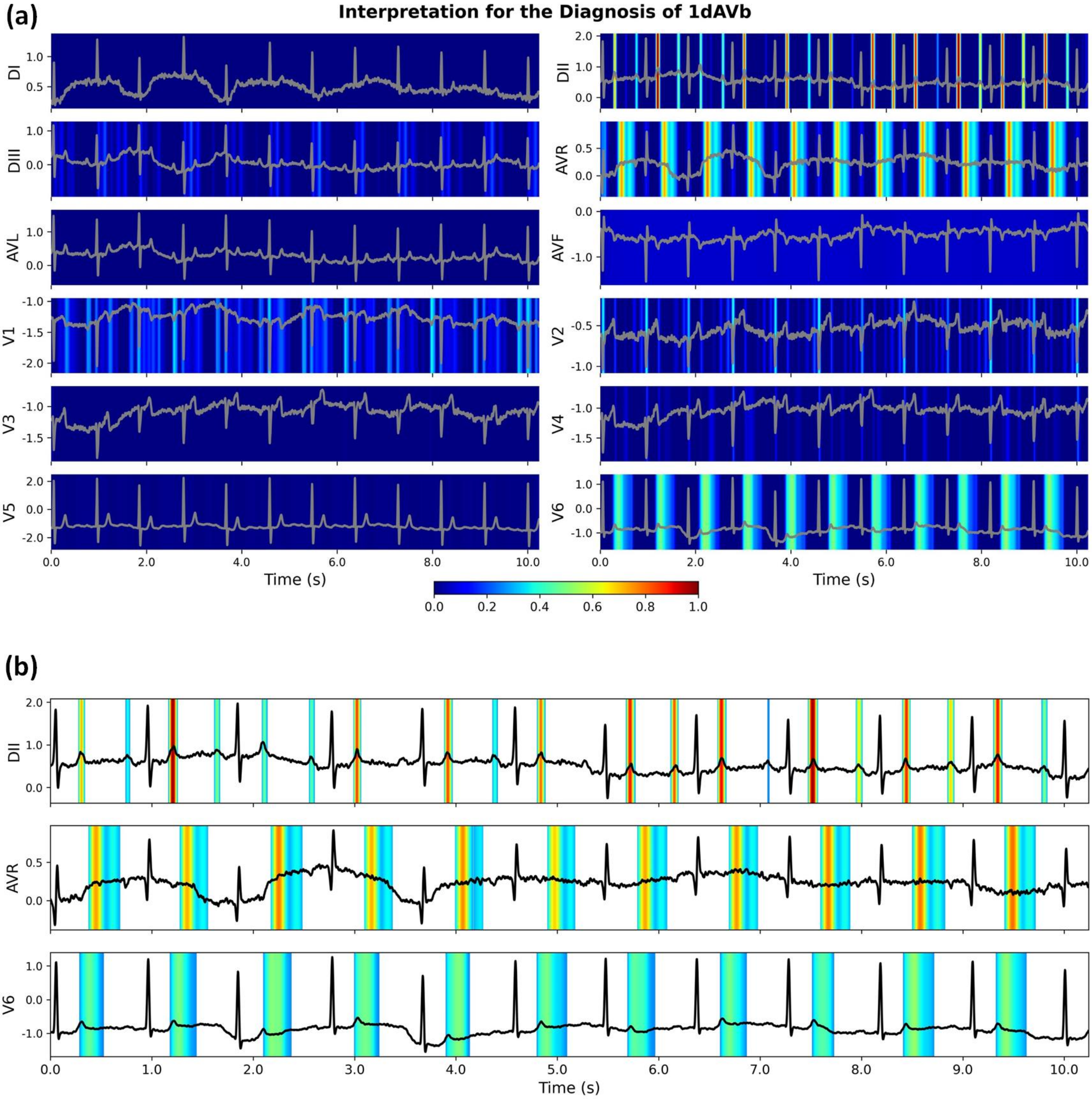

Extended Figure S5: Interpretation for the diagnosis of first degree atrioventricular block (1dAVb) using the developed DNN model. **(a)** The original calculated heatmaps for 12 ECG leads, with colour bar ranging from blue to red indicating the increasing weights of importance. **(b)** The refined view of heatmaps for DII, AVR, and V6 leads with background colour removed. For the diagnosis of 1dAVb, the criteria include prolonged PR interval, normal QRS, and normal rhythm [8]. Because the QRS complex is normal, our developed DNN model highlights other morphologies for the diagnosis, such as the T and P waves in the DII lead, and the segments after T waves in the AVR and V6 leads. Using the combination of salient features, the DNN model has a probability of 0.932 to diagnose the 1dAVb with the ECG recording.

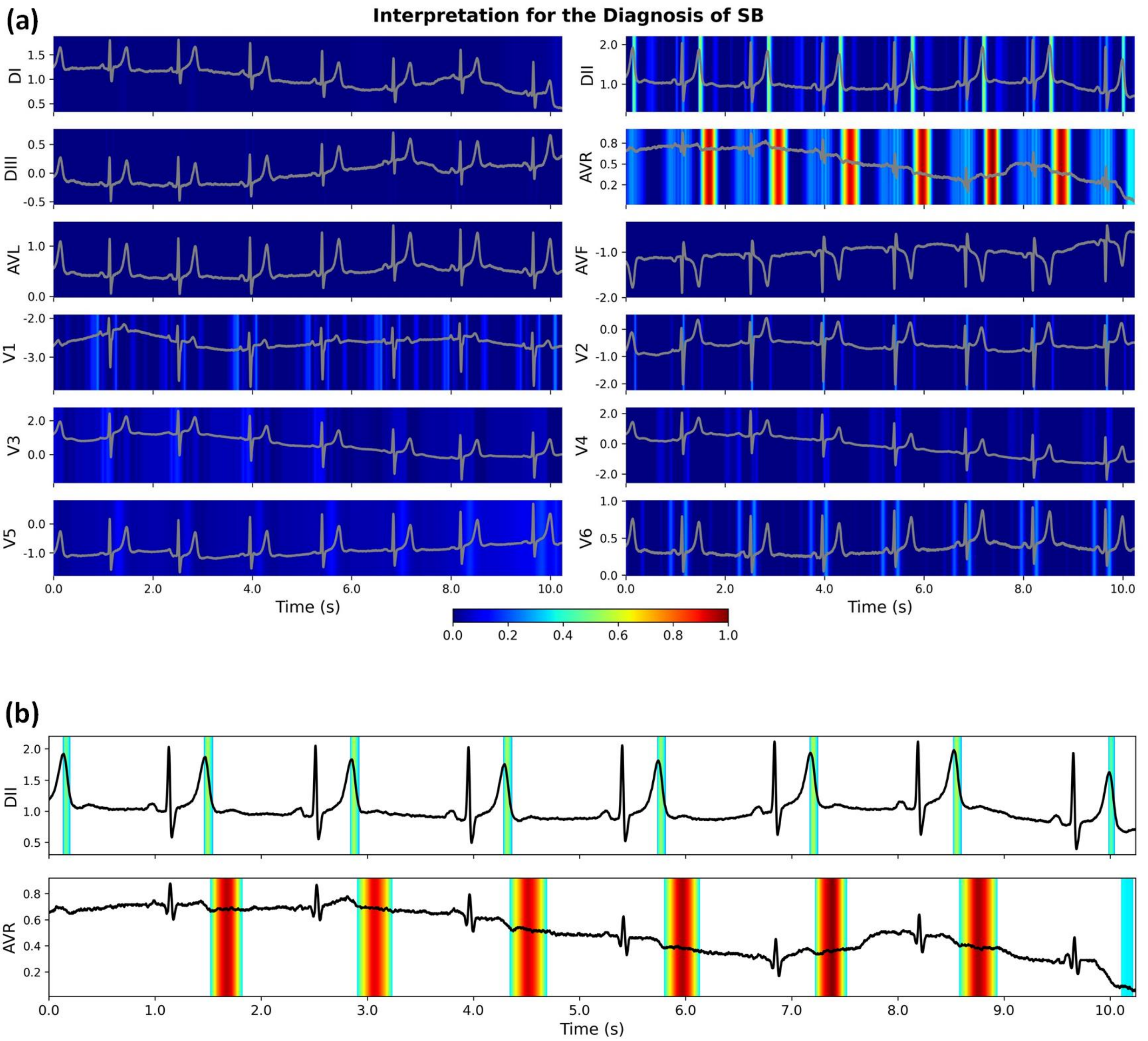

Extended Figure S6: Interpretation for the diagnosis of sinus bradycardia (SB) using the developed DNN model. **(a)** The original calculated heatmaps for 12 ECG leads, with colour bar ranging from blue to red indicating the increasing weights of importance. **(b)** The refined view of heatmaps for the DII and AVR leads with background colour removed. SB is defined as a sinus rate below 50 bpm with otherwise normal P, QRS and T waves [8]; while the prominent U waves were also reported for asymptomatic SB in literature [17]. For the diagnosis of SB, our developed DNN model highlights the U waves in the AVR lead and the downslops of T waves in the DII lead. Using the combination of salient features, the DNN model has a probability of 0.932 to diagnose the SB using the ECG recording.

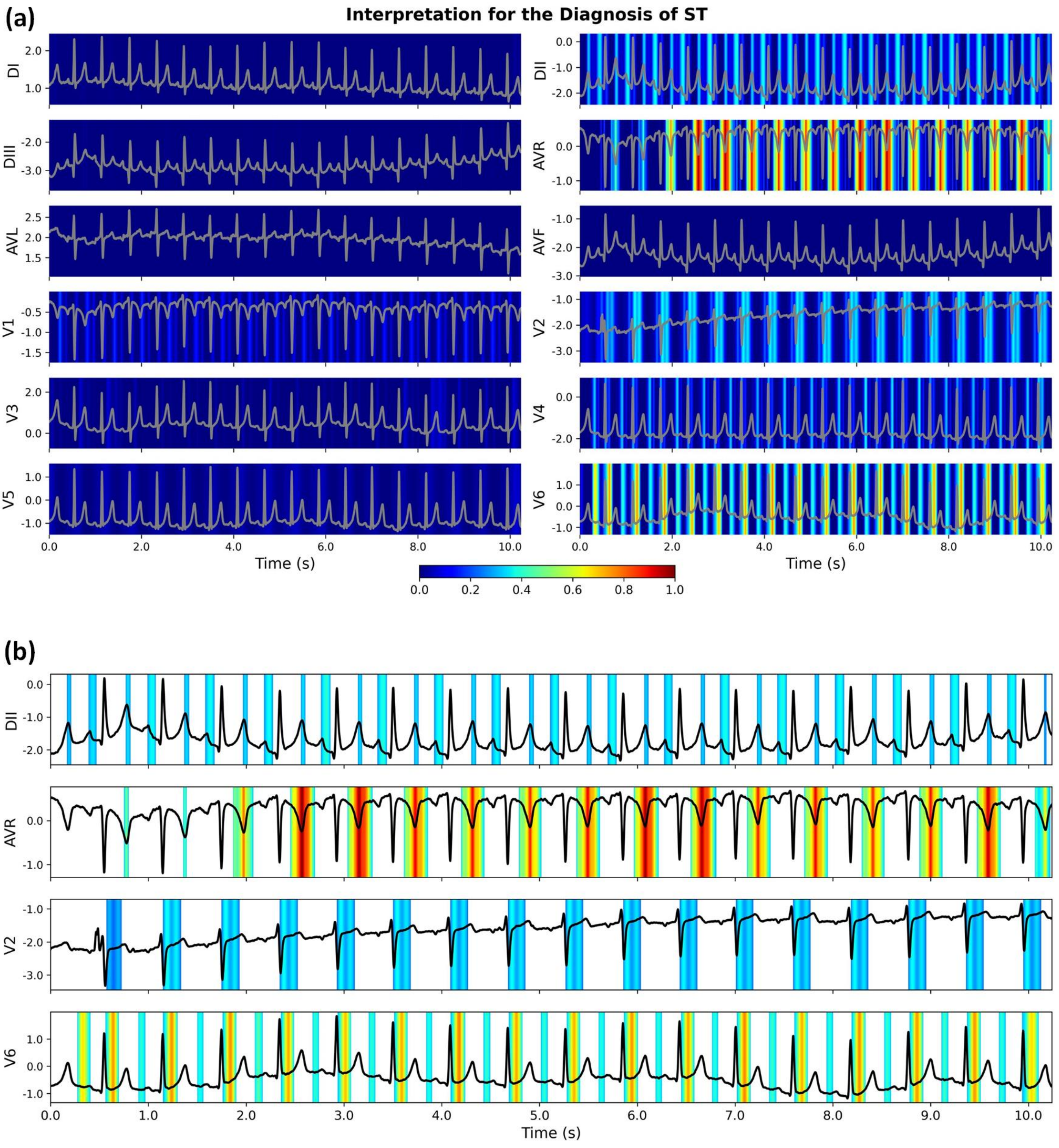

Extended Figure S7: Interpretation for the diagnosis of sinus tachycardia (ST) using our developed DNN model. **(a)** The original calculated heatmaps for 12 ECG leads, with colour bar ranging from blue to red indicating the increasing weights of importance. **(b)** The refined view of heatmaps for the DII, AVR, V2, and V6 leads with background colour removed. ST is the sinus rhythm with a heart rate greater than 100 bpm, and it has normal P wave preceding every QRS complex [8]. For the diagnosis of ST, our developed DNN model highlights the downslops of T and P waves in the DII lead, T waves in the AVR lead, and ST segments in the V2 and V6 leads. Using the combination of salient features, the DNN model has a probability of 0.948 to diagnose the ST with the ECG recording.

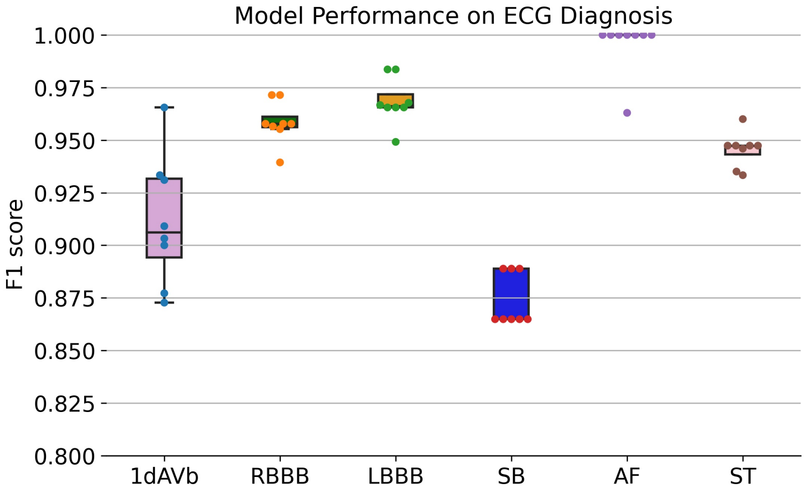

Extended Figure S8: Model performance on the diagnosis of ECG abnormalities, and the F1 scores were obtained by running the model with eight different random seeds.

Extended Table S3: Model performance of hyperparameter tuning and ablation study for ECG diagnosis.

Model Types	1dAVb	RBBB	LBBB	SB	AF	ST	micro_avg
Conv_Filters_16_16_16_48	0.696	0.909	0.966	0.842	0.923	0.960	0.893
Conv_Filters_32_16_32_48	0.816	0.971	0.929	0.889	0.880	0.907	0.906
Kernel_Sizes_16_16_48_16	0.783	0.930	0.983	0.842	0.917	0.960	0.914
Kernel_Sizes_16__8_48_48	0.793	0.986	0.949	0.865	0.960	0.947	0.920
n_Conv_Block_2_16_16_48_48	0.750	0.928	0.949	0.909	0.960	0.923	0.904
Res_Blocks_2_16_16_48_48	0.800	0.882	0.949	0.865	1.000	0.960	0.908
Dense_Integ._16_16_48_48	0.889	0.919	0.951	0.821	0.889	0.909	0.904
BiLSTM_Integ._16_16_48_48	0.867	0.957	0.983	0.839	1.000	0.911	0.926
BiLSTM_Dense_16_16_48_48	0.906	0.909	0.949	0.889	1.000	0.946	0.930

* The table shows the model performance (F1 scores) of hyperparameter tuning and ablation study for ECG diagnosis, with the models trained on a subset of 300K ECGs from the whole CODE dataset. The different types of models are read as follows: 'Conv_Filters_16_16_16_48' indicates the investigation of the numbers of convolutional (Conv) filters, each of the first two residual blocks has 16 filters with a kernel size of 16, and each of rest two residual blocks has 16 filters with a kernel size of 48; 'Kernel_Sizes_16_16_48_16' indicates the investigation of the kernel sizes of convolutional filters, each of the first two residual blocks has 16 filters with a kernel size of 16, and each of rest two residual blocks has 48 filters with a kernel size of 16; 'n_Conv_Block_2_16_16_48_48' indicates that the model has 2 Conv layers in each of the four residual blocks; Conv layers in the first two residual blocks have 16 filters with a kernel size of 16, and have 48 filters with a kernel size of 48 for the rest two residual blocks; 'Res_Blocks_2_16_16_48_48' indicates that the model has 2 residual blocks; The first block has 16 filters with a kernel size of 16, and the second residual blocks has 48 filters with a kernel size of 48; 'Dense_Integ._16_16_48_48' indicates the ablation study of investigating the architecture of feature integration, and the model only uses dense layers for feature integration; 'BiLSTM_Integ._16_16_48_48' indicates the ablation study of investigating the architecture of feature integration, and the model only uses LSTM layers for feature integration; 'BiLSTM_Dense_16_16_48_48' indicates the ablation study of investigating the architecture of feature integration, this is the model that was used in this study; The model has four residual blocks with four convolutional layers for each block, and it uses both LSTM and dense layers for feature integration. The micro average (micro_avg) in the table computes the score across different classes, and it calculates the total true positives, false negatives, and false positives to obtain a comprehensive metric for performance evaluation.

Extended Table S4: Performance comparison for the diagnosis of ECG abnormalities with the McNemar's test.

	McNemar's χ^2 test (p-value)					
	1dAVb	RBBB	LBBB	SB	AF	ST
DNN *vs* Cardio. Rd.	4.923 (**0.027**)	1.500 (0.221)	0.250 (0.617)	0.000 (1.000)	4.167 (**0.041**)	0.444 (0.505)
DNN *vs* Emerg. Rd.	12.500 (**0.000**)	3.273 (0.070)	0.167 (0.683)	0.125 (0.724)	5.143 (**0.023**)	0.000 (1.000)
DNN *vs* Medical Sd.	12.191 (**0.001**)	0.800 (0.371)	0.125 (0.724)	0.444 (0.505)	8.100 (**0.004**)	0.900 (0.343)
DNN *vs* Cardio. #1	4.083 (**0.043**)	0.000 (1.000)	0.000 (1.000)	0.125 (0.724)	1.333 (0.248)	0.083 (0.773)
DNN *vs* Cardio. #2	0.167 (0.683)	0.500 (0.480)	1.333 (0.248)	0.125 (0.724)	1.333 (0.248)	0.100 (0.752)
DNN *vs* A.H.R. [2]	1.125 (0.289)	0.500 (0.480)	1.333 (0.248)	0.000 (1.000)	1.333 (0.248)	0.500 (0.480)

*The table shows the two-sided McNemar's χ^2 test [2, 18, 19] and p-values (bracketed values) for the performance comparison between our developed DNN model and other evaluation results, including Cardio. Rd.: the 4th year cardiology residents; Emerg. Rd.: 3rd year emergency residents; Medical Sd.: 5th year medical students; Cardio. #1: the 1st cardiologist; Cardio. #2: the 2nd cardiologist; A.H.R.: the state-of-the-art benchmark model developed in [2]. The bold-faced values denote statistical significance ($p < 0.05$) for the comparison of paired evaluation results.

Extended Table S5: Performance comparison for the diagnosis of ECG abnormalities with Cohen's kappa coefficient.

	Cohen's kappa coefficient					
	1dAVb	RBBB	LBBB	SB	AF	ST
DNN *vs* Cardio. Rd.	0.737	0.915	0.926	0.869	0.766	0.864
DNN *vs* Emerg. Rd.	0.716	0.819	0.889	0.785	0.691	0.930
DNN *vs* Medical Sd.	0.699	0.926	0.857	0.751	0.700	0.855
DNN *vs* Cardio. #1	0.792	0.956	0.909	0.760	0.868	0.816
DNN *vs* Cardio. #2	0.889	0.970	0.947	0.760	0.887	0.855
DNN *vs* A.H.R. [2]	0.862	0.972	0.947	0.921	0.868	0.972

*The table shows Cohen's kappa coefficients [15] for the performance comparison between our developed DNN model and other evaluation results, including Cardio. Rd.: 4th year cardiology residents; Emerg. Rd.: 3rd year emergency residents; Medical Sd.: 5th year medical students; Cardio. #1: the 1st cardiologist; Cardio. #2: the 2nd cardiologist; A.H.R.: the state-of-the-art benchmark model developed in [2]. The Cohen's kappa coefficient is used to calculate the inter-rater agreement between paired measures, with a value closer to 1 indicating a higher agreement between the two measures.

Extended Table S6: Model performance on the external dataset retrieved from the PhysioNet/CinC 2017 Challenge [20].

	Precision	Recall	F1-score	Support
AF	0.885	0.979	0.929	$n = 47$
Normal	0.869	0.939	0.903	$n = 148$
Other rhythms	0.933	0.646	0.764	$n = 65$
Noise	0.972	0.875	0.921	$n = 40$
micro_avg	0.894	0.873	0.884	$n = 300$
macro_avg	0.915	0.860	0.879	$n = 300$
weighted_avg	0.899	0.873	0.879	$n = 300$

*The PhysioNet dataset contains ECG recordings with varied lengths of data points. We either truncate or zero pad the ECG recordings to match with those in the Brazilian CODE ECG dataset. Each resulted ECG recording has a total of 4,096 data points with a sampling frequency of 300 Hz. We test the model on the holdout benchmark validation dataset, as the competition was closed and the standard testing dataset is not publicly available. The competition reported the best performance of an average F1-score of 0.83 [21]. In comparison, our model has an average F1-score of 0.884. The micro average (micro_avg) in the table computes the score across different classes, and it calculates the total true positives, false negatives, and false positives to obtain a comprehensive metric; The macro average (macro_avg) is defined as the arithmetic mean of all scores of different classes; The weighted average (weighted_avg) computes the score considering the proportion of samples in each class.

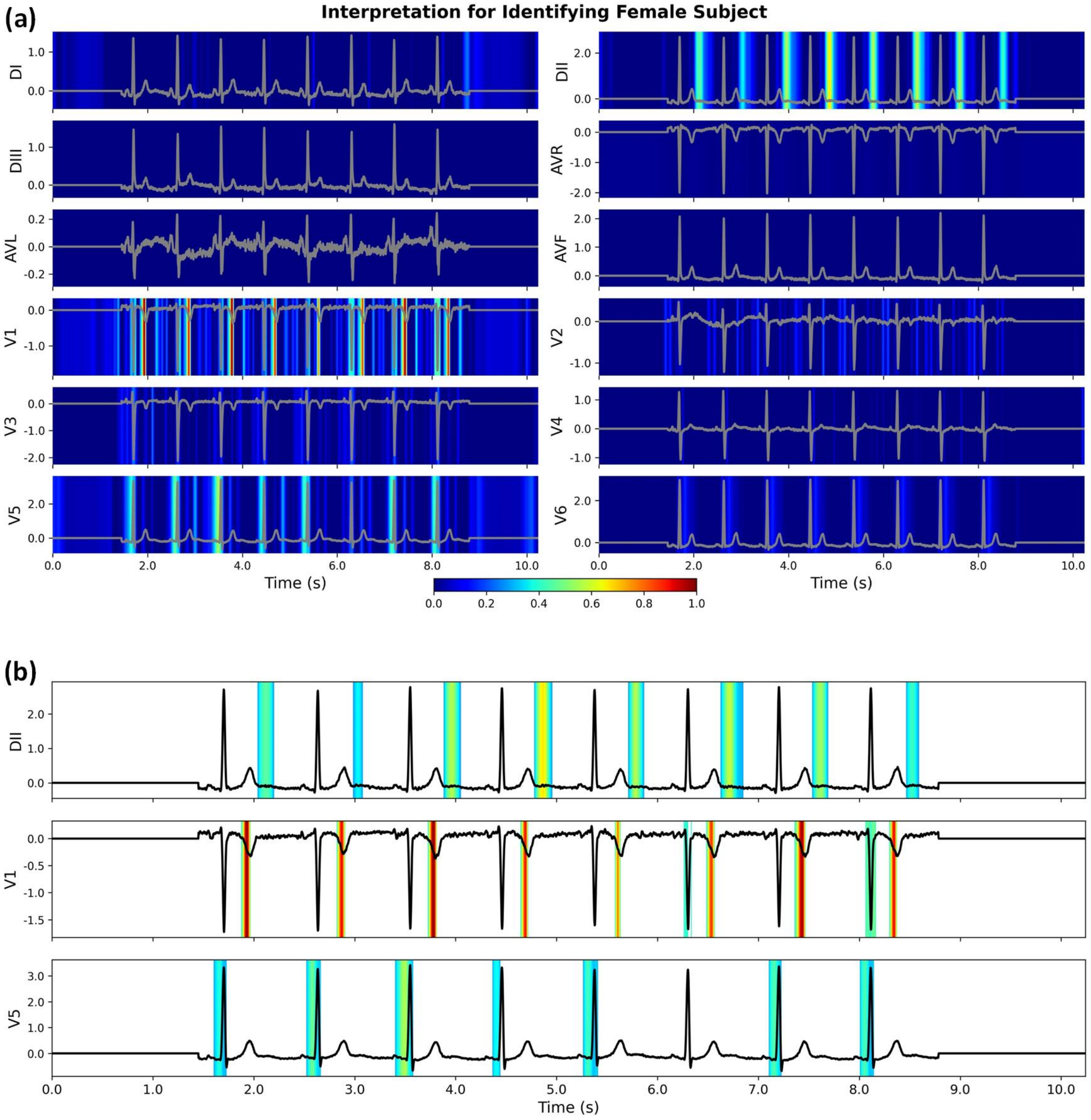

Extended Figure S9: Interpretation for identifying female subject using our developed DNN model. **(a)** The original calculated heatmaps for 12 ECG leads, with colour bar ranging from blue to red indicating the increasing weights of importance. **(b)** The refined view of the DII, V1, and V5 leads with background colour removed. Using the combination of salient features, the DNN model has a probability of 0.971 to identify gender for the female subject using the ECG recording.

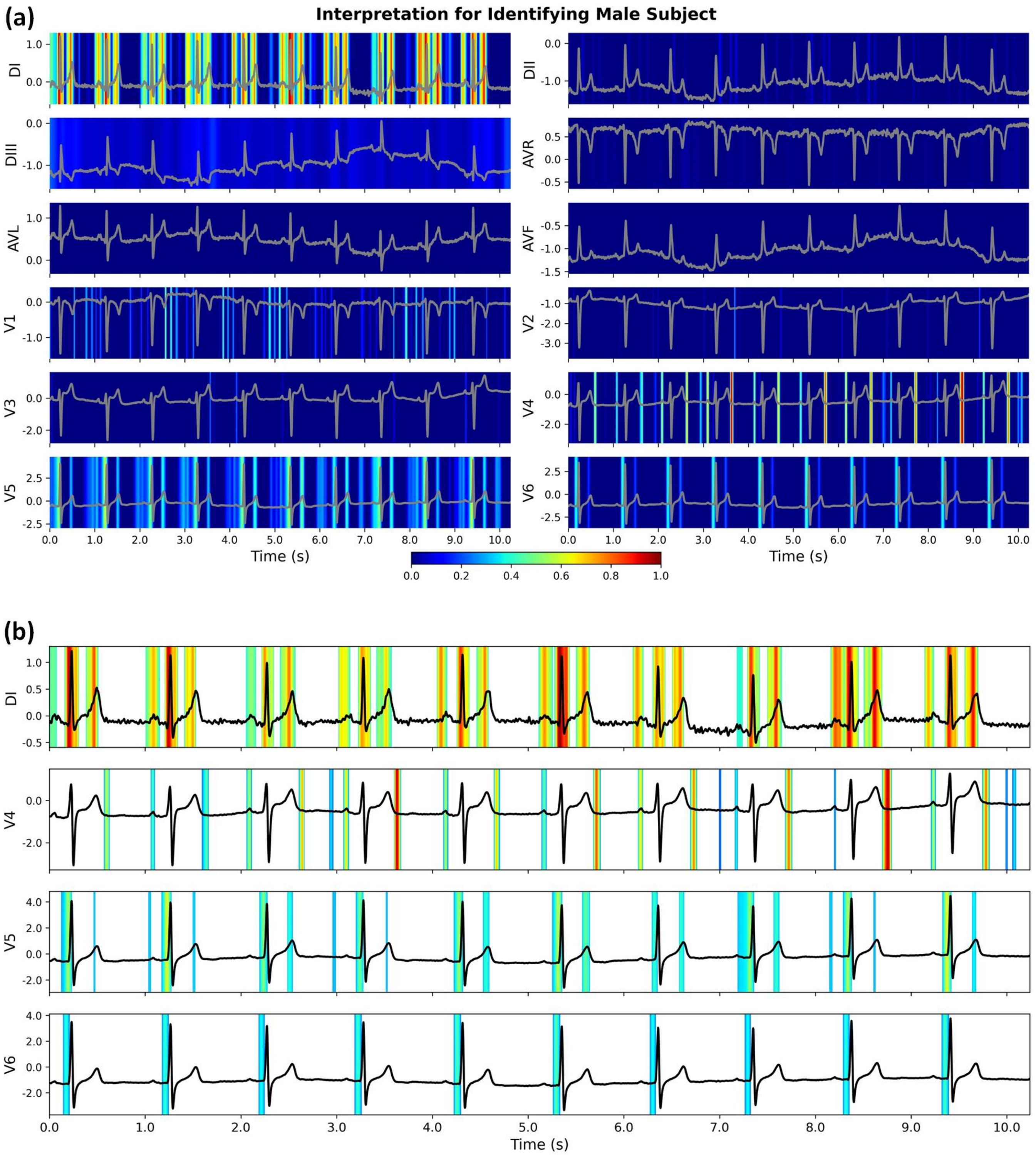

Extended Figure S10: Interpretation for identifying male subject using our developed DNN model. **(a)** The original calculated heatmaps for 12 ECG leads, with colour bar ranging from blue to red indicating the increasing weights of importance. **(b)** The refined view of the DI, V4, V5, and V6 leads with background colour removed. Using the combination of salient features, the DNN model has a probability of 0.981 to identify gender for the male subject using the ECG recording.

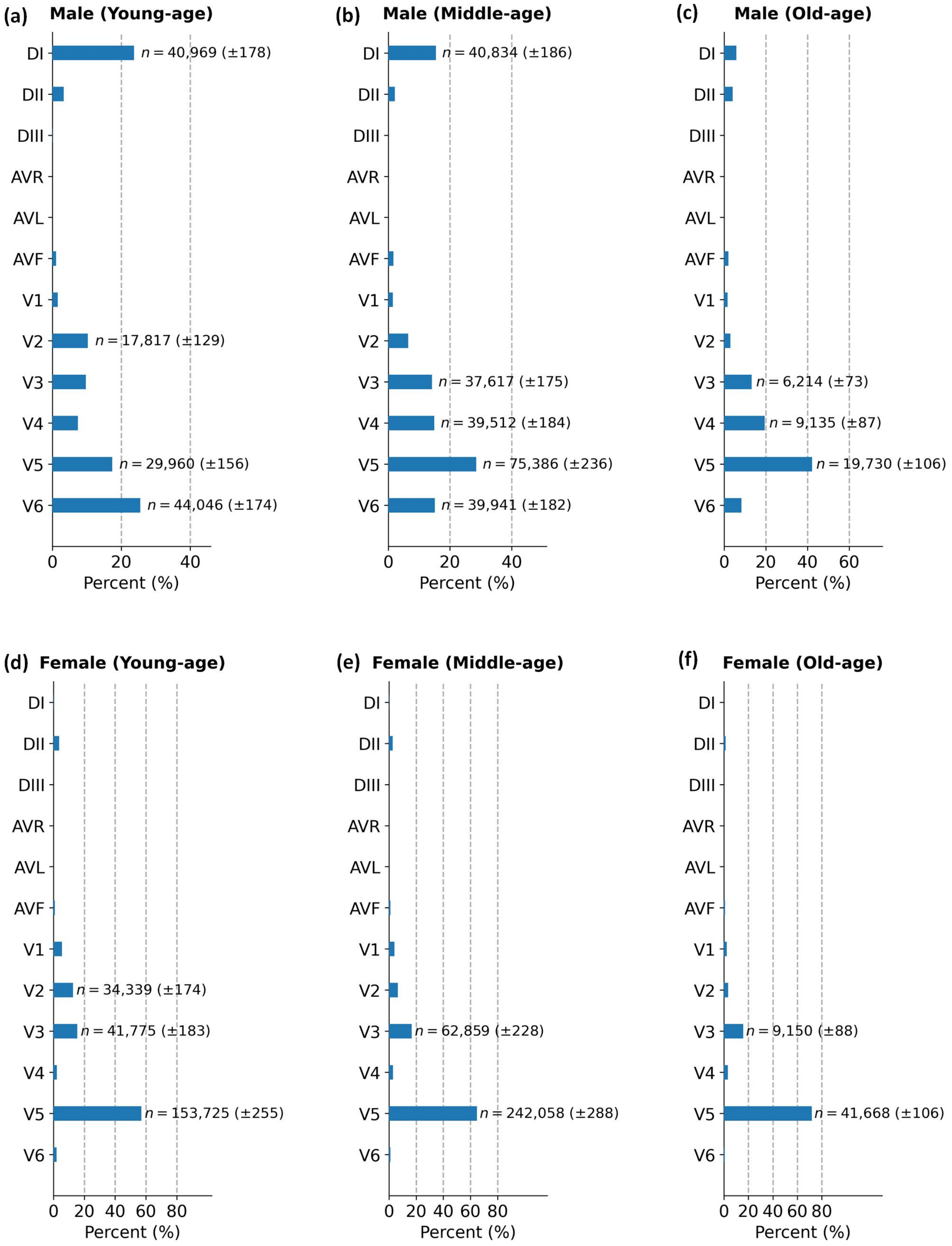

Extended Figure S11: Distributions of dominant ECG leads for gender identification using our developed DNN model. **(a)** Distribution for young-age male subjects ($yr < 45$). **(b)** Distribution for middle-age male subjects ($45 \leq yr < 75$). **(c)** Distribution for old-age male subjects ($yr \geq 75$). **(d)** Distribution for young-age female subjects. **(e)** Distribution for middle-age female subjects. **(f)** Distribution for old-age female subjects. We annotate the number of occurrences when the dominant lead accounts for more than 10% of all the 12 ECG leads. The number of occurrences is presented as mean and standard deviation.

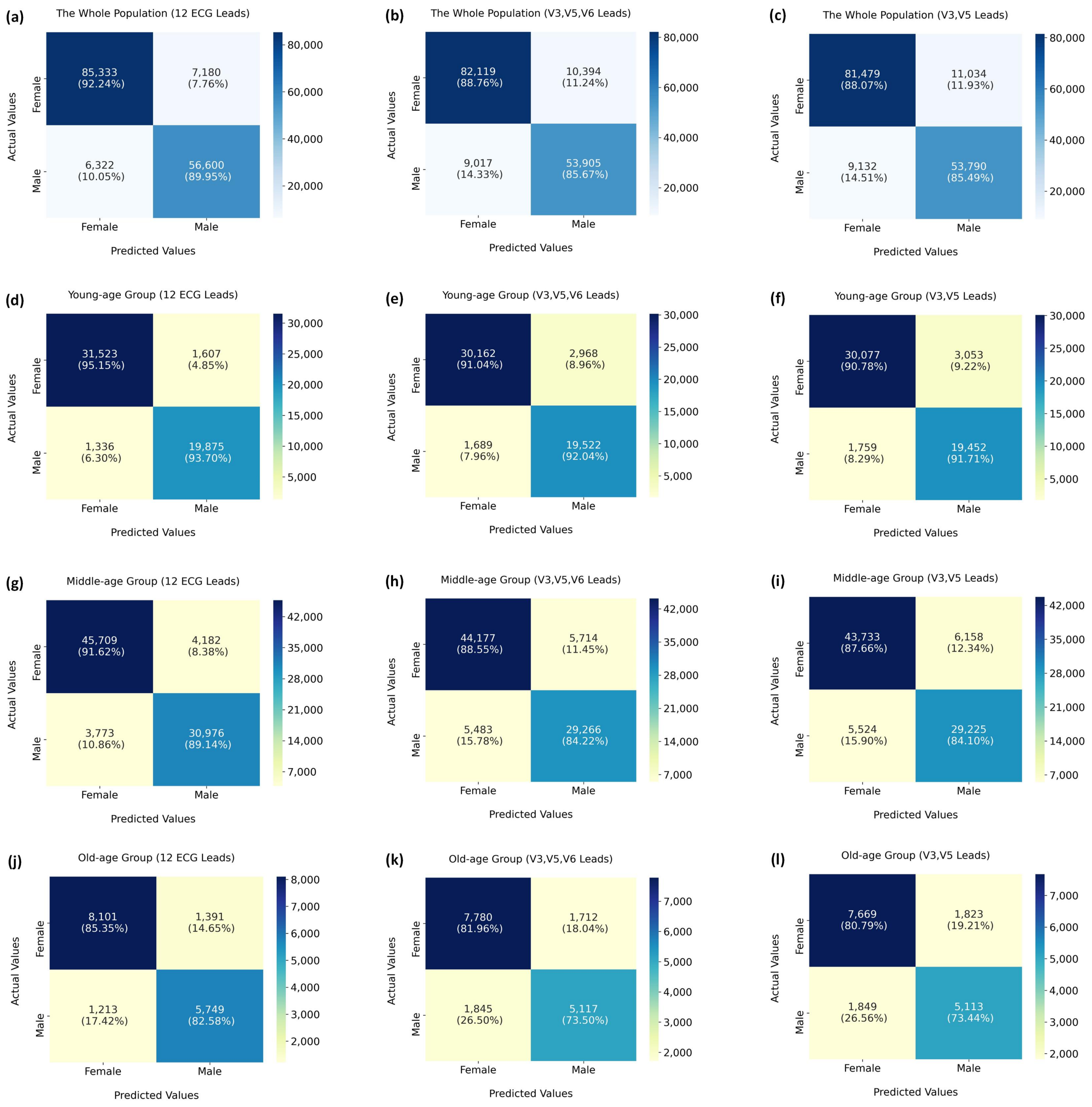

Extended Figure S12: Confusion matrices of gender identification for the DNN model using 12 ECG leads, dominant V3, V5, and V6 leads, and dominant V3 and V5 leads. **(a)-(c)** Performance comparison in the whole population. **(d)-(f)** Performance comparison in the young-age group ($yr < 45$). **(g)-(i)** Performance comparison in the middle-age group ($45 \leq yr < 75$). **(j)-(l)** Performance comparison in the old-age group ($yr \geq 75$).

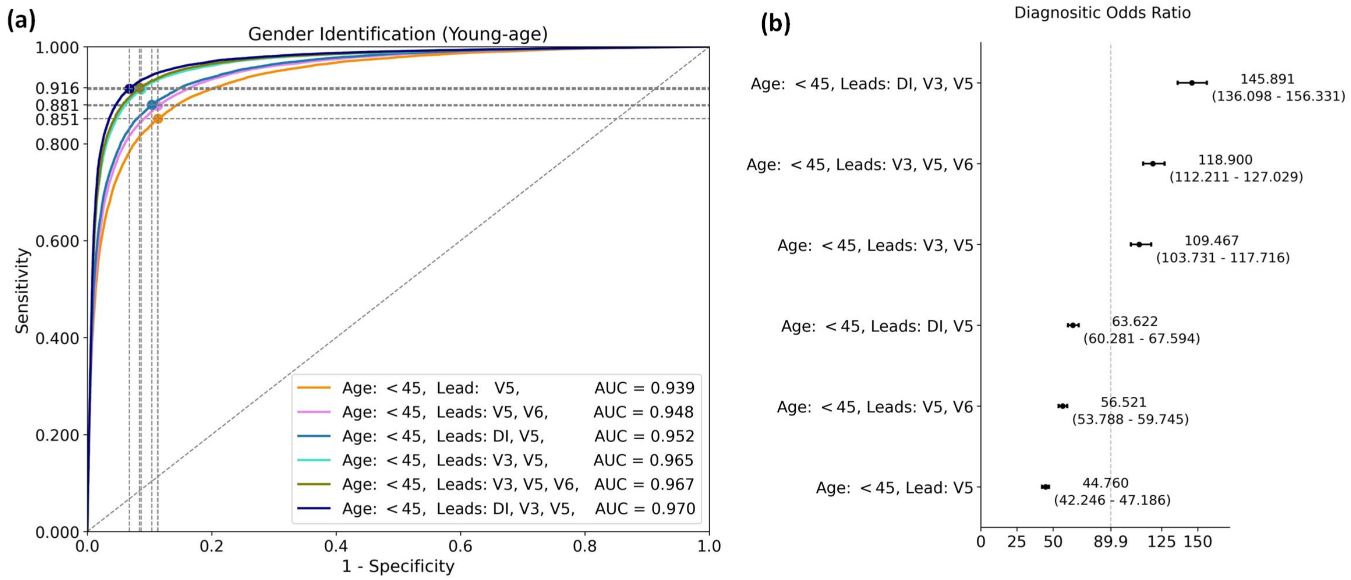

Extended Figure S13: Model performance on gender identification for young-age group ($yr < 45$) using dominant ECG leads. **(a)** The ROC and AUC scores for gender identification using different combinations of dominant leads. **(b)** The distribution of DOR values (95% CI) for model performance on gender identification using different combinations of dominant leads.

Extended Table S7: Performance comparison of gender identification for young-age group ($yr < 45$) using dominant leads.

Dominant ECG Leads	Sensitivity (95% CI)	Specificity (95% CI)	AUC Score (95% CI)
Lead: V5	0.851 (0.845-0.878)	0.886 (0.860-0.893)	0.939 (0.936-0.940)
Leads: V5, V6	0.878 (0.871-0.884)	0.887 (0.881-0.894)	0.948 (0.946-0.950)
Leads: DI, V5	0.881 (0.876-0.891)	0.896 (0.886-0.901)	0.952 (0.950-0.953)
Leads: V3, V5	0.912 (0.899-0.924)	0.914 (0.902-0.927)	0.965 (0.964-0.967)
Leads: V3, V5, V6	0.916 (0.909-0.920)	0.916 (0.912-0.923)	0.967 (0.965-0.968)
Leads: DI, V3, V5	0.914 (0.910-0.930)	0.932 (0.918-0.936)	0.970 (0.969-0.972)

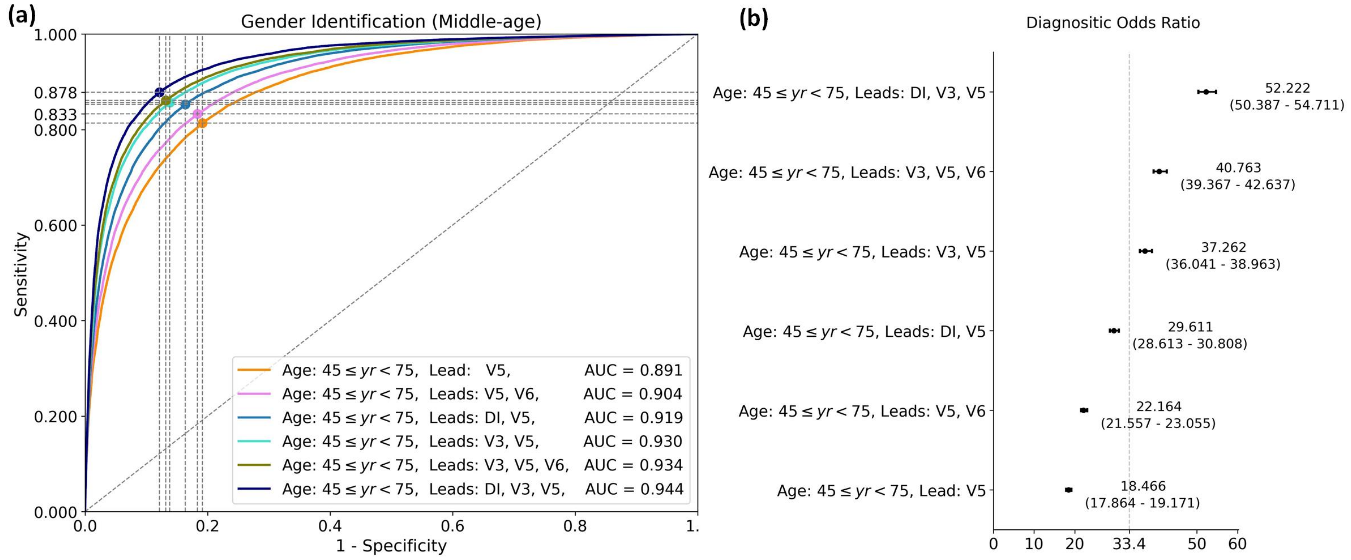

Extended Figure S14: Model performance of gender identification for middle-age group ($45 \leq yr < 75$) using dominant ECG leads. **(a)** The ROC and AUC scores for gender identification using different combinations of dominant leads. **(b)** The distribution of DOR values (95% CI) for model performance on gender identification using different combinations of dominant leads.

Extended Table S8: Performance comparison of gender identification for middle-age group ($45 \leq yr < 75$) using dominant ECG leads.

Dominant ECG Leads	Sensitivity (95% CI)	Specificity (95% CI)	AUC Score (95% CI)
Lead: V5	0.814 (0.807-0.822)	0.809 (0.801-0.815)	0.891 (0.888-0.893)
Leads: V5, V6	0.833 (0.805-0.836)	0.817 (0.815-0.844)	0.904 (0.902-0.906)
Leads: DI, V5	0.853 (0.835-0.860)	0.837 (0.829-0.854)	0.919 (0.917-0.921)
Leads: V3, V5	0.857 (0.844-0.867)	0.862 (0.852-0.875)	0.930 (0.928-0.932)
Leads: V3, V5, V6	0.861 (0.848-0.872)	0.868 (0.858-0.882)	0.934 (0.933-0.936)
Leads: DI, V3, V5	0.878 (0.869-0.886)	0.879 (0.871-0.888)	0.944 (0.942-0.945)

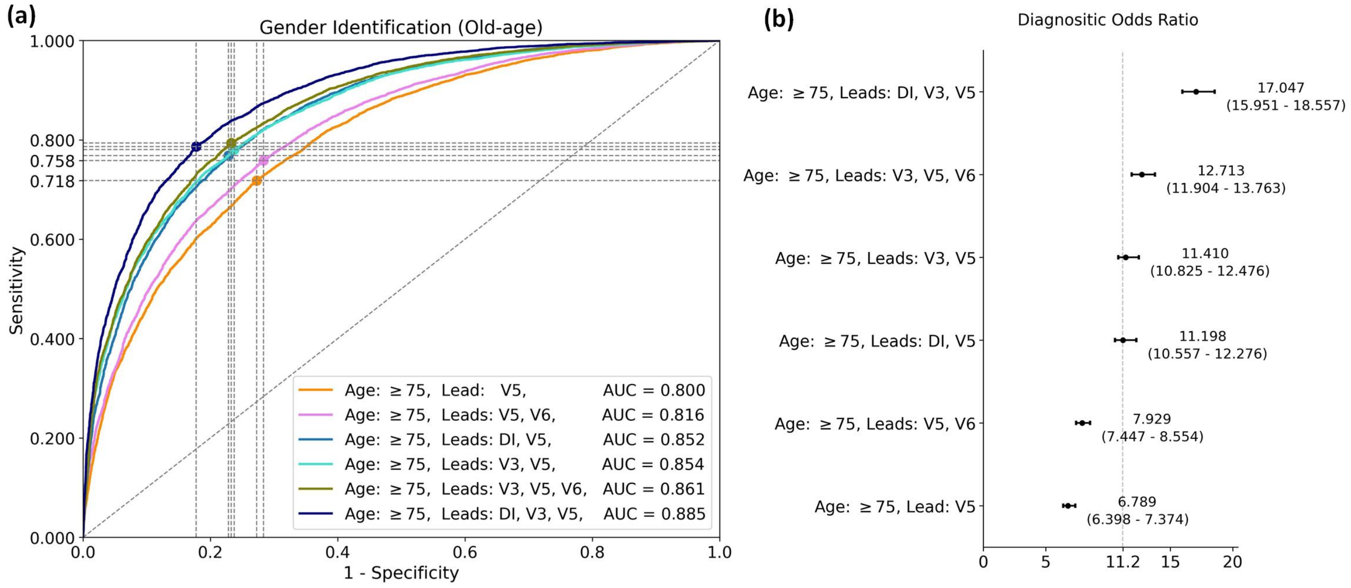

Extended Figure S15: Model performance of gender identification for old-age group ($yr > 75$) using dominant ECG leads. **(a)** The ROC and AUC scores for gender identification using different combinations of dominant leads. **(b)** The distribution of DOR values (95% CI) for model performance on gender identification using different combinations of dominant leads.

Extended Table S9: Performance comparison of gender identification for old-age group ($yr \geq 75$) using dominant leads.

Dominant ECG Leads	Sensitivity (95% CI)	Specificity (95% CI)	AUC Score (95% CI)
Lead: V5	0.718 (0.705-0.753)	0.727 (0.694-0.741)	0.800 (0.793-0.806)
Leads: V5, V6	0.758 (0.716-0.777)	0.716 (0.700-0.759)	0.816 (0.809-0.822)
Leads: DI, V5	0.768 (0.756-0.820)	0.771 (0.723-0.784)	0.852 (0.846-0.858)
Leads: V3, V5	0.780 (0.732-0.800)	0.763 (0.745-0.813)	0.854 (0.849-0.860)
Leads: V3, V5, V6	0.794 (0.762-0.803)	0.767 (0.759-0.798)	0.861 (0.856-0.866)
Leads: DI, V3, V5	0.786 (0.781-0.834)	0.822 (0.777-0.828)	0.885 (0.880-0.890)

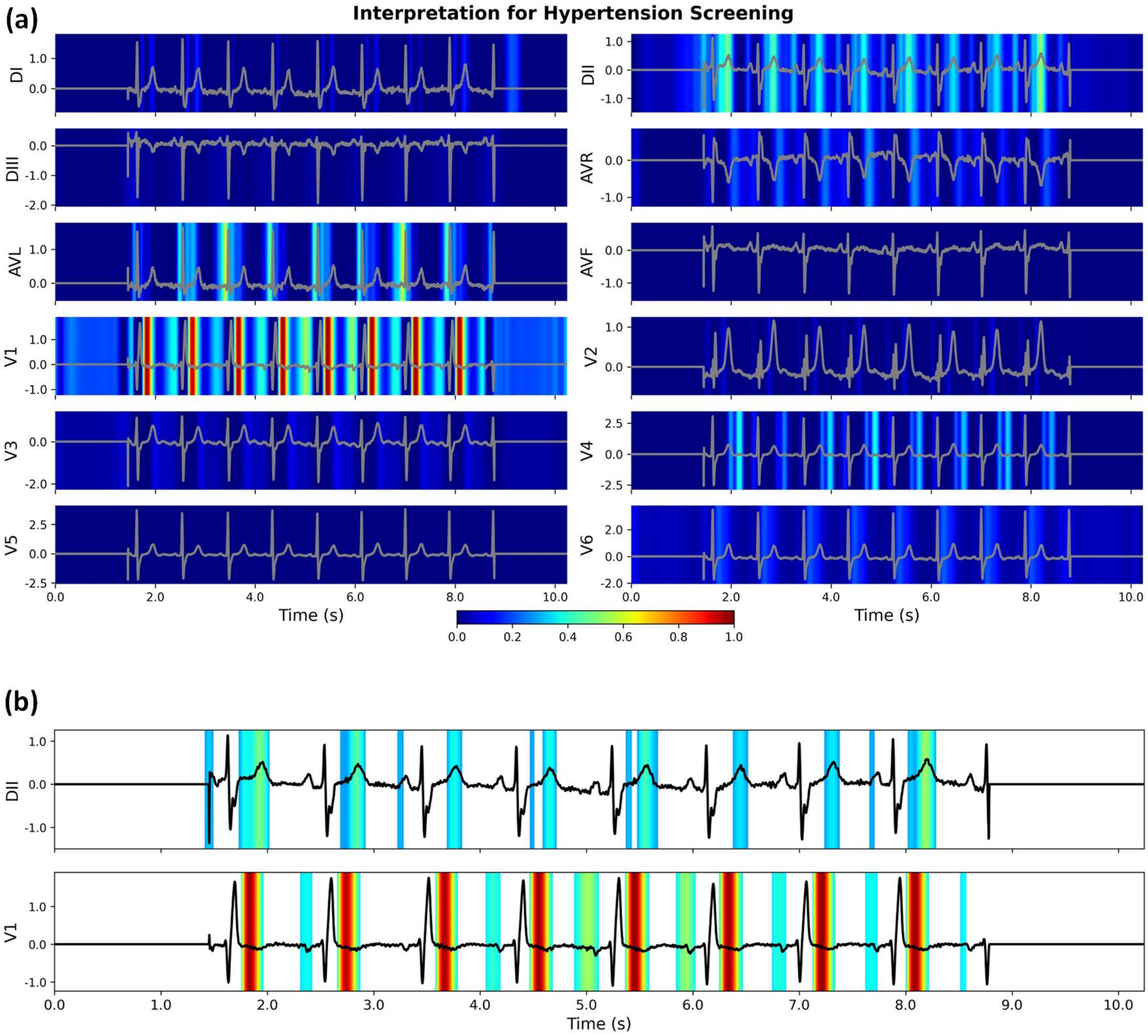

Extended Figure S16: Interpretation for hypertension screening using our developed DNN model. **(a)** The original calculated heatmaps for 12 ECG leads, with colour bar ranging from blue to red indicating the increasing weights of importance. **(b)** The refined view of the DII and V1 leads with background colour removed. Using the combination of salient features, our DNN model has a probability of 0.902 to screen hypertension for the subject using the ECG recording.

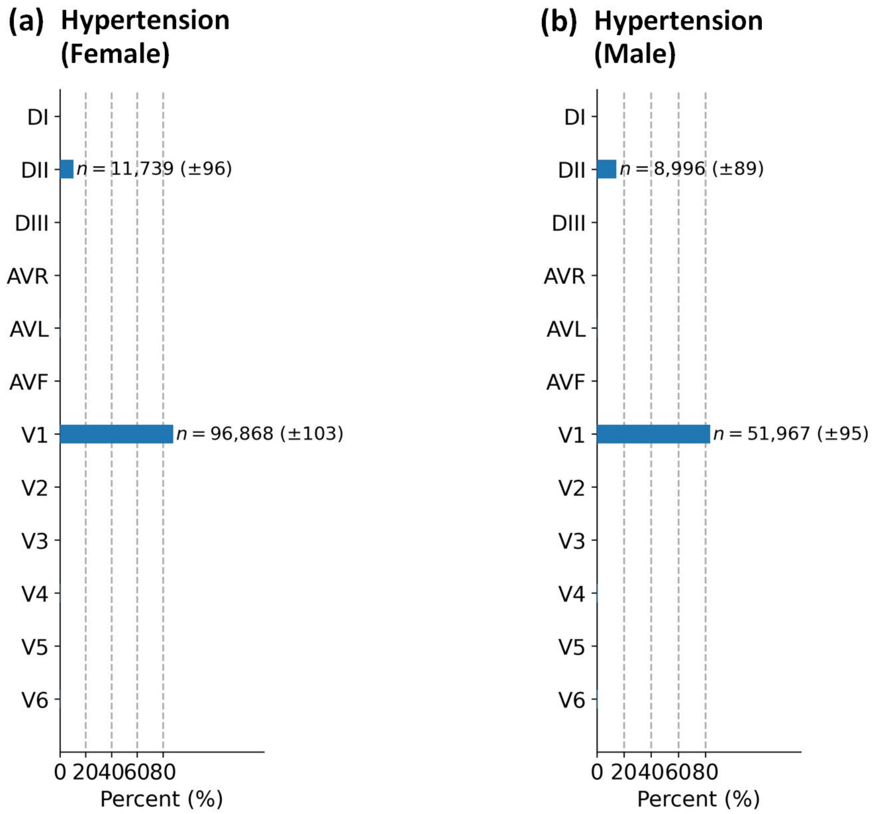

Extended Figure S17: Distributions of dominant ECG leads for hypertension screening using our developed DNN model. **(a)** Distribution of dominant leads for female subjects. **(b)** Distribution of dominant leads for male subjects. We annotate the number of occurrences when the dominant lead accounts for more than 10% of all the 12 ECG leads. The number of occurrences is presented as mean and standard deviation.

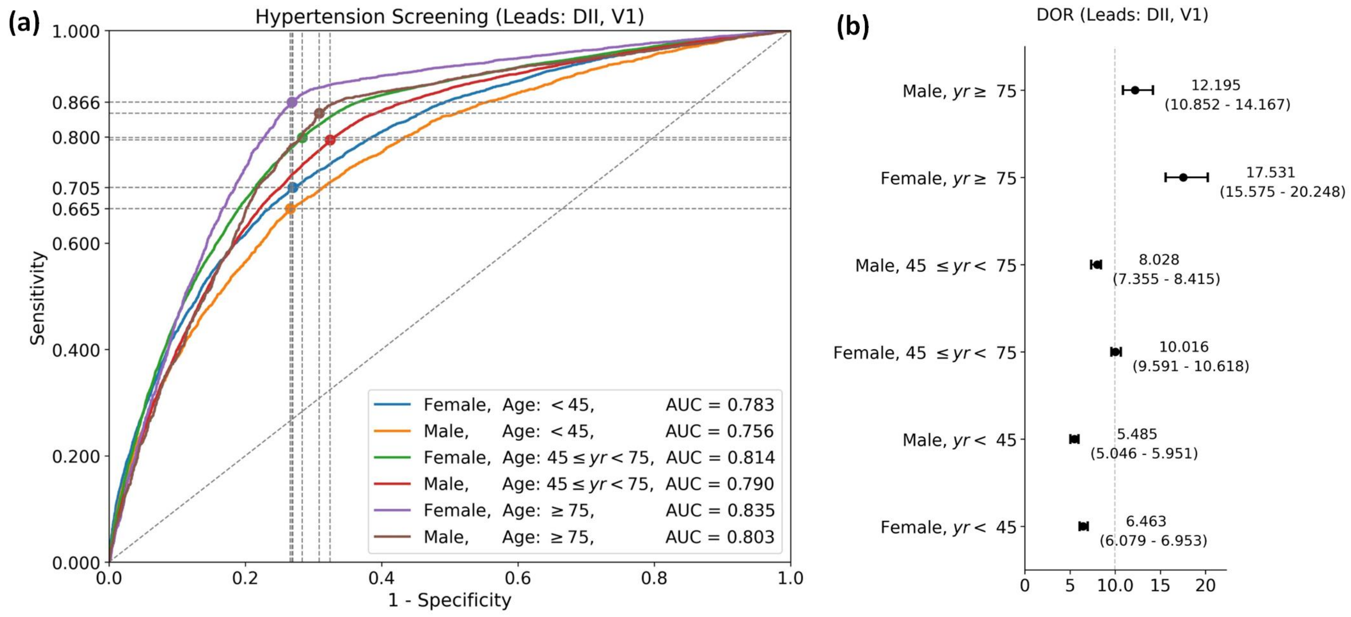

Extended Figure S18: Model performance on hypertension screening in different populations using the dominant DII and V1 leads. **(a)** The ROC and AUC scores for hypertension screening in different populations. **(b)** The distribution of DOR values (95% CI) for model performance on hypertension screening in different populations.

Extended Table S10: Performance comparison for hypertension screening using dominant DII and V1 ECG leads in terms of age and gender differences.

Population Groups	Sensitivity (95% CI)	Specificity (95% CI)	AUC Score (95% CI)
Female, $yr < 45$	0.705 (0.682-0.728)	0.730 (0.709-0.753)	0.783 (0.776-0.790)
Male, $yr < 45$	0.665 (0.650-0.718)	0.734 (0.677-0.742)	0.756 (0.747-0.765)
Female, $45 \leq yr < 75$	0.799 (0.781-0.814)	0.716 (0.703-0.735)	0.814 (0.810-0.818)
Male, $45 \leq yr < 75$	0.794 (0.748-0.801)	0.676 (0.670-0.718)	0.790 (0.786-0.795)
Female, $yr \geq 75$	0.866 (0.844-0.885)	0.731 (0.713-0.751)	0.835 (0.827-0.844)
Male, $yr \geq 75$	0.845 (0.822-0.866)	0.692 (0.672-0.712)	0.803 (0.793-0.814)

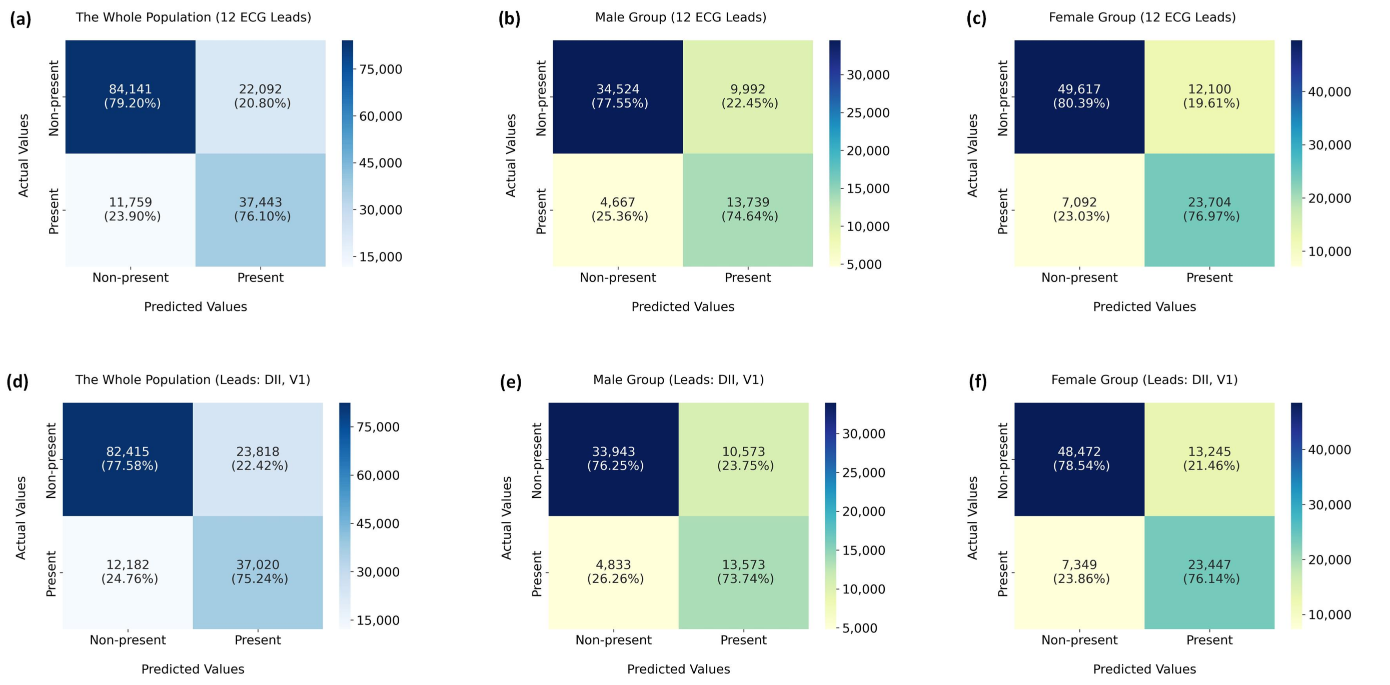

Extended Figure S19: Confusion matrices for hypertension screening using 12 ECG leads, and the dominant DII and V1 leads. **(a)**-**(c)** Performance comparison of hypertension screening using 12-lead ECGs in different populations. **(d)**-**(f)** Performance comparison of hypertension screening using dominant ECG leads in different populations.

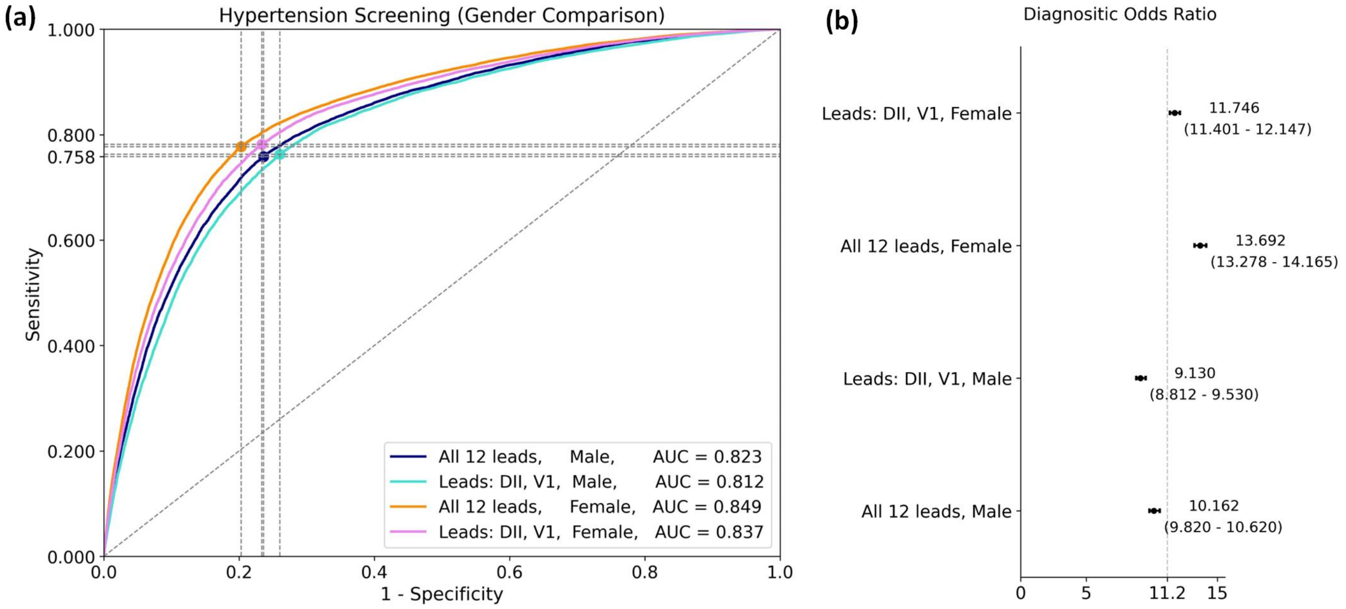

Extended Figure S20: Model performance on hypertension screening using 12 ECG leads and dominant ECG leads in terms of gender differences. **(a)** The ROC and AUC scores for hypertension screening using 12 ECG leads and dominant ECG leads. **(b)** The distribution of DOR values (95% CI) for model performance on hypertension screening using 12 ECG leads and dominant ECG leads.

Extended Table S11: Performance comparison on hypertension screening using different ECG leads in terms of gender differences.

ECG Leads & Gender Differences	Sensitivity (95% CI)	Specificity (95% CI)	AUC Score (95% CI)
All 12 leads, Male	0.758 (0.745-0.772)	0.764 (0.752-0.778)	0.823 (0.820-0.827)
Leads: DII, V1, Male	0.763 (0.744-0.775)	0.740 (0.729-0.760)	0.812 (0.808-0.816)
All 12 leads, Female	0.777 (0.769-0.791)	0.797 (0.784-0.805)	0.849 (0.847-0.852)
Leads: DII, V1, Female	0.781 (0.765-0.787)	0.767 (0.763-0.783)	0.837 (0.834-0.840)

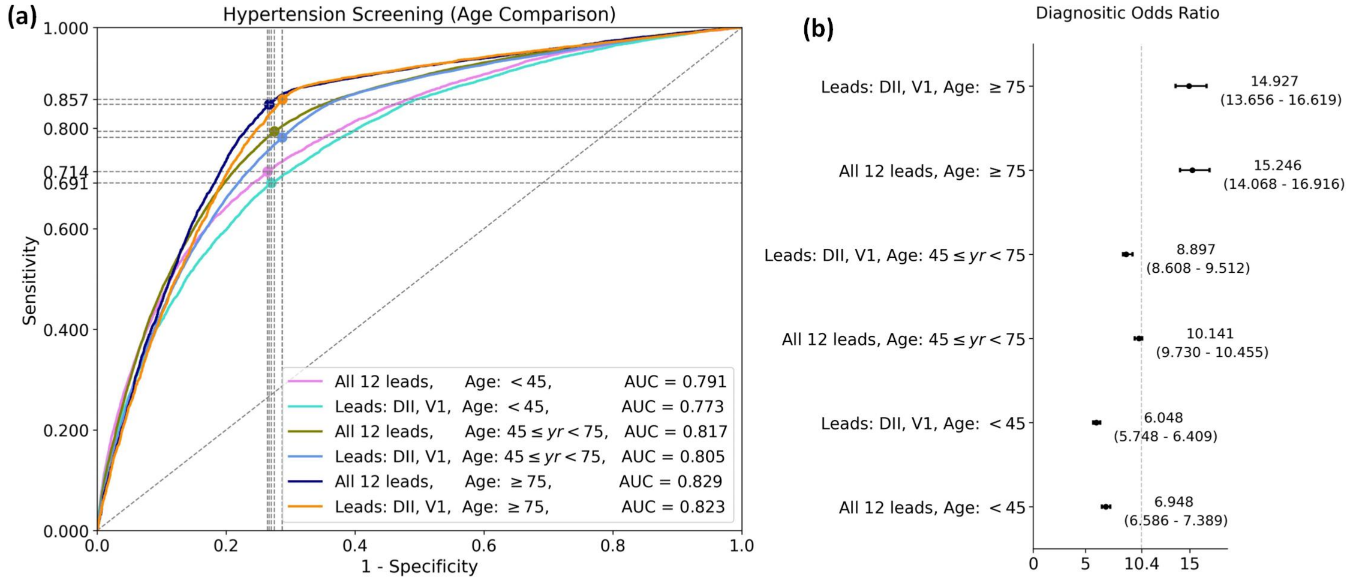

Extended Figure S21: Model performance on hypertension screening using 12 ECG leads and dominant ECG leads in terms of age differences. **(a)** The ROC and AUC scores for hypertension screening using 12 ECG leads and dominant ECG leads. **(b)** The distribution of DOR values (95% CI) for model performance on hypertension screening using 12 ECG leads and dominant ECG leads.

Extended Table S12: Performance comparison on hypertension screening using different ECG leads in terms of age differences.

ECG Leads & Age Differences	Sensitivity (95% CI)	Specificity (95% CI)	AUC Score (95% CI)
All 12 leads, Age: < 45	0.714 (0.687-0.736)	0.736 (0.717-0.763)	0.791 (0.785-0.796)
Leads: DII, V1, Age: < 45	0.691 (0.678-0.711)	0.730 (0.710-0.740)	0.773 (0.767-0.779)
All 12 leads, Age: $45 \leq yr < 75$	0.794 (0.774-0.798)	0.725 (0.721-0.744)	0.817 (0.814-0.820)
Leads: DII, V1, Age: $45 \leq yr < 75$	0.782 (0.770-0.809)	0.713 (0.690-0.724)	0.805 (0.802-0.808)
All 12 leads, Age: ≥ 75	0.847 (0.831-0.865)	0.733 (0.717-0.749)	0.829 (0.822-0.836)
Leads: DII, V1, Age: ≥ 75	0.857 (0.844-0.874)	0.713 (0.697-0.726)	0.823 (0.816-0.829)

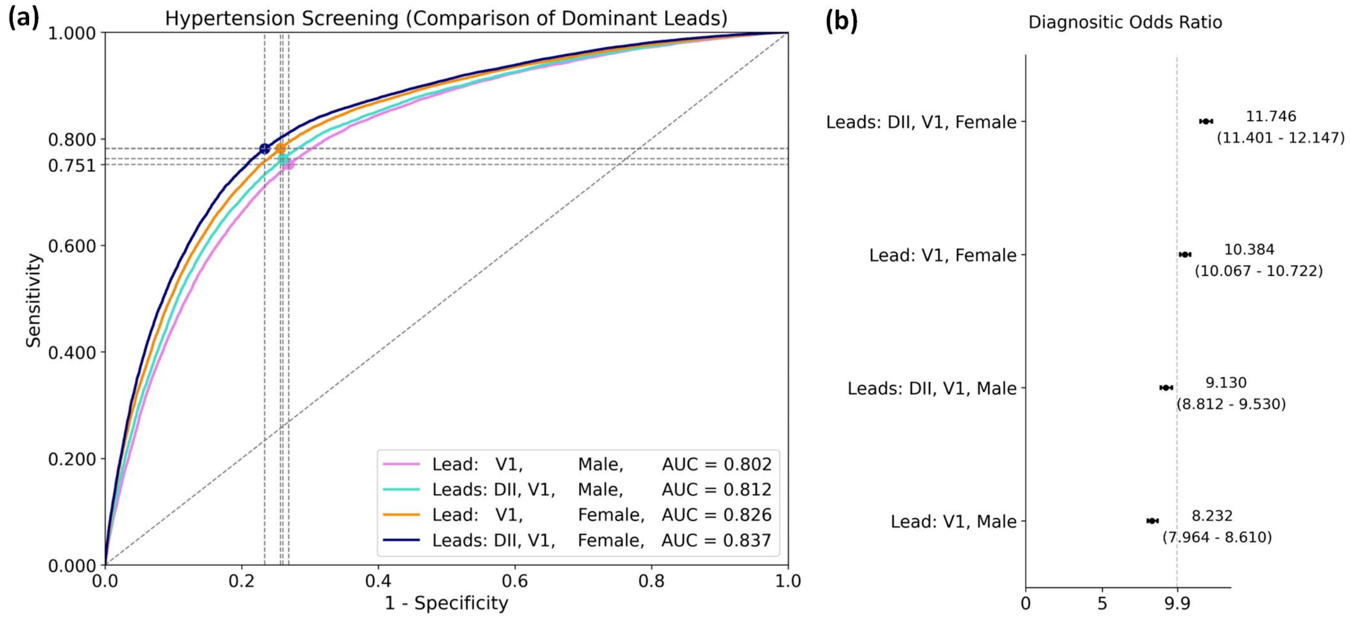

Extended Figure S22: Model performance on hypertension screening using different combinations of dominant ECG leads in terms of gender differences. (**a**) The ROC and AUC scores for hypertension screening using different dominant ECG leads. (**b**) The distribution of DOR values (95% CI) for model performance on hypertension screening using different dominant ECG leads.

Extended Table S13: Performance comparison for hypertension screening using different dominant ECG leads in terms of gender differences.

ECG Leads & Gender Differences	Sensitivity (95% CI)	Specificity (95% CI)	AUC Score (95% CI)
Lead: V1, Male	0.751 (0.738-0.764)	0.731 (0.719-0.746)	0.802 (0.798-0.806)
Leads: DII, V1, Male	0.763 (0.744-0.775)	0.740 (0.729-0.760)	0.812 (0.808-0.816)
Lead: V1, Female	0.782 (0.751-0.793)	0.743 (0.733-0.773)	0.826 (0.823-0.829)
Leads: DII, V1, Female	0.781 (0.765-0.787)	0.767 (0.763-0.783)	0.837 (0.834-0.840)

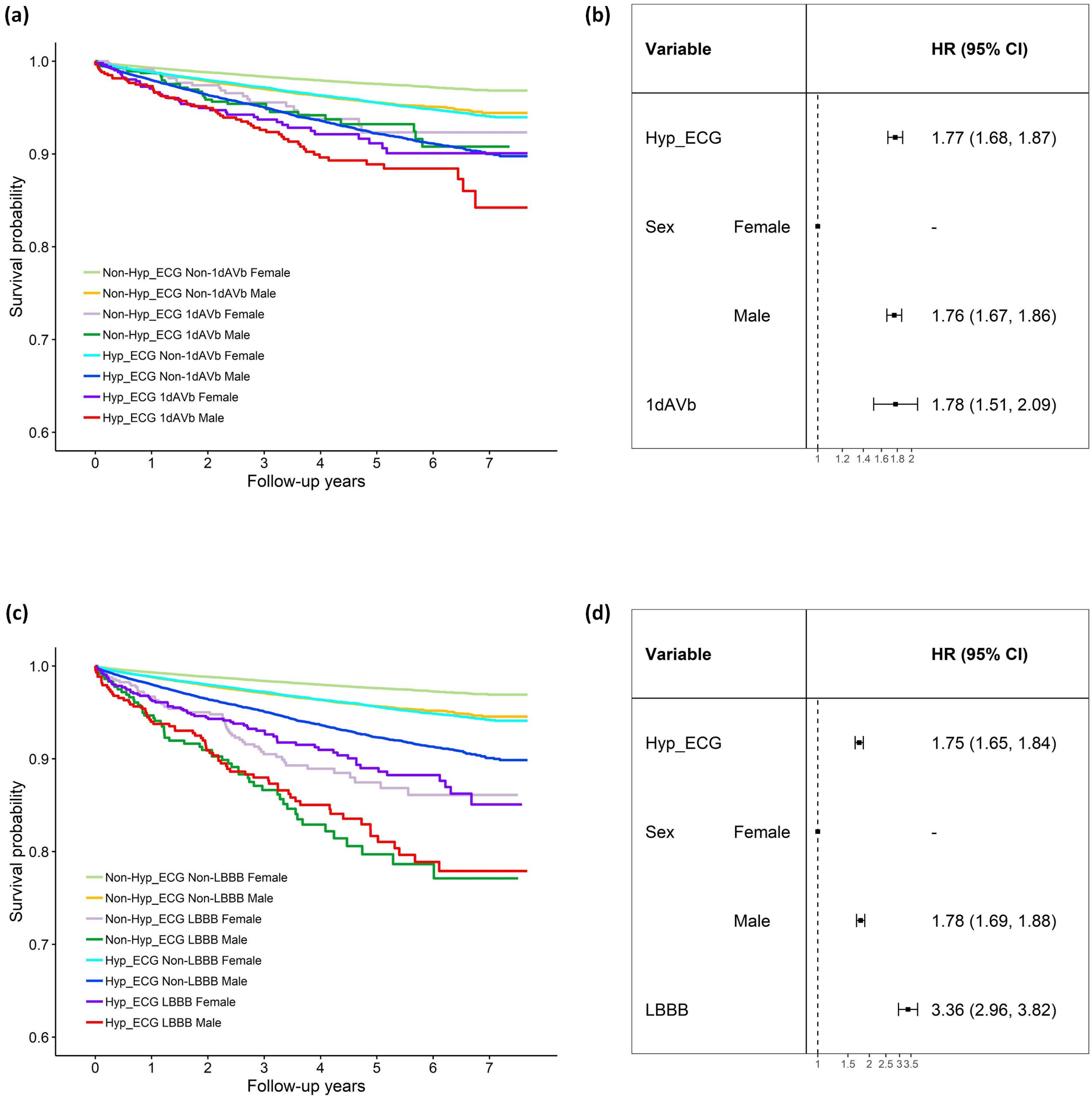

Extended Figure S23: Mortality risk stratification. (**a**) shows the Kaplan–Meier survival curves for different cohorts, which demonstrate the impacts of hypertension, 1dAVb, and gender differences. (**b**) provides HRs for mortality risk adjusted by hypertension, 1dAVb, and gender. (**c**) shows the Kaplan–Meier survival curves for different cohorts; The results indicate the impacts of hypertension and LBBB on mortality having gender differences, and LBBB has high risks in both males and females regardless the presence of hypertension. (**d**) shows HRs for the risk of mortality adjusted for hypertension, LBBB, and gender (Hyp_ECG: ECG-predicted hypertension using 12 leads).

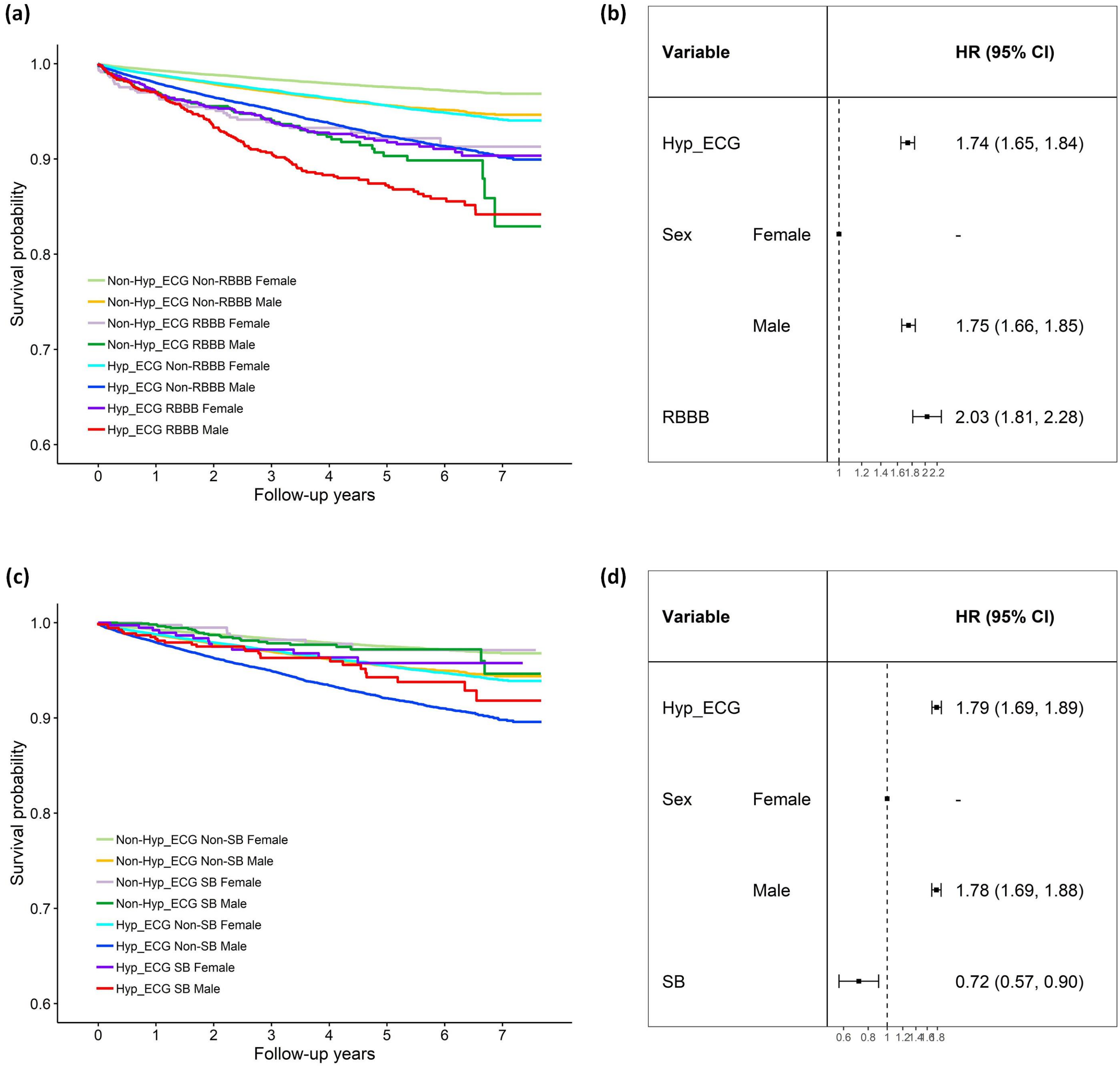

Extended Figure S24: Mortality risk stratification. **(a)** shows the Kaplan–Meier survival curves for different cohorts. The results indicate the impacts of hypertension and RBBB on mortality having gender differences; **(b)** provides HRs for the risk of mortality adjusted for hypertension, RBBB, and gender. **(c)** shows the Kaplan–Meier survival curves for different cohorts, indicating the impacts from ECG-predicted hypertension, SB, and gender differences. **(d)** provides HRs for the risk of mortality adjusted for hypertension, SB, and gender. (Hyp_ECG: ECG-predicted hypertension using 12 leads.)

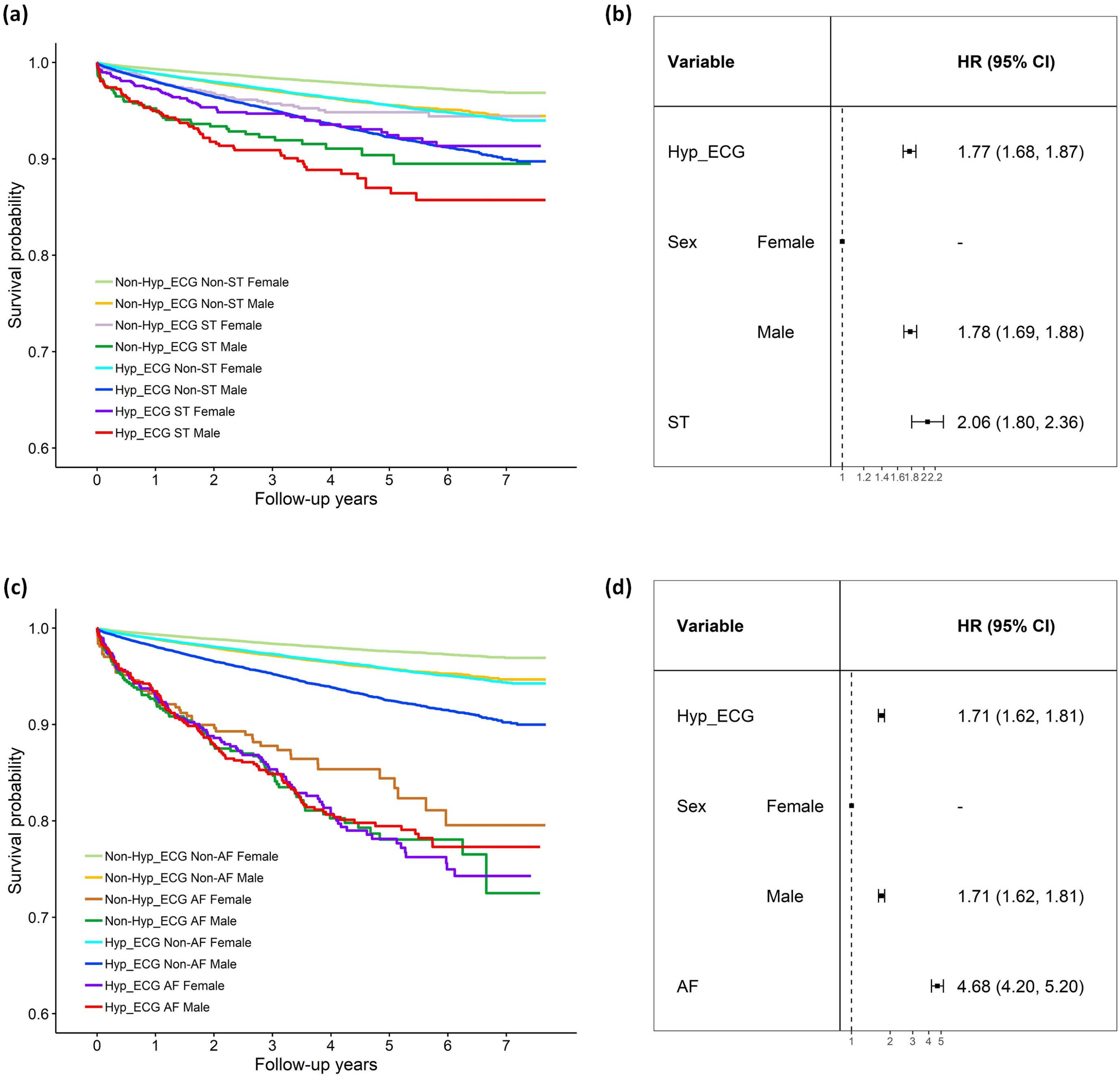

Extended Figure S25: Mortality risk stratification. **(a)** shows the Kaplan–Meier survival curves for different cohorts; The results indicate the impacts of hypertension and ST on mortality having gender differences. **(b)** provides HRs for the risk of mortality adjusted for hypertension, ST, and gender. **(c)** shows the Kaplan–Meier survival curves for different cohorts, indicating the impacts from ECG-predicted hypertension, AF, and gender differences. **(d)** provides HRs for the risk of mortality adjusted for hypertension, AF, and gender. In particular, we observed the high risk of mortality for cohorts with AF regardless the presence of hypertension. (Hyp_ECG: ECG-predicted hypertension using 12 leads)

References

[1] Antonio Luiz P Ribeiro, Gabriela MM Paixão, Paulo R Gomes, Manoel Horta Ribeiro, Antonio H Ribeiro, Jessica A Canazart, Derick M Oliveira, Milton P Ferreira, Emilly M Lima, Jermana Lopes de Moraes, et al. Tele-electrocardiography and bigdata: the CODE (Clinical Outcomes in Digital Electrocardiography) study. *Journal of Electrocardiology*, 57:S75–S78, 2019.

[2] Antônio H Ribeiro, Manoel Horta Ribeiro, Gabriela MM Paixão, Derick M Oliveira, Paulo R Gomes, Jéssica A Canazart, Milton PS Ferreira, Carl R Andersson, Peter W Macfarlane, Wagner Meira Jr, et al. Automatic diagnosis of the 12-lead ECG using a deep neural network. *Nature Communications*, 11(1):1–9, 2020.

[3] Emilly M Lima, Antônio H Ribeiro, Gabriela MM Paixão, Manoel Horta Ribeiro, Marcelo M Pinto Filho, Paulo R Gomes, Derick M Oliveira, Ester C Sabino, Bruce B Duncan, Luana Giatti, et al. Deep neural network estimated electrocardiographic-age as a mortality predictor. *Nature Communications*, 12:5117, 2021.

[4] Ramprasaath R Selvaraju, Michael Cogswell, Abhishek Das, Ramakrishna Vedantam, Devi Parikh, and Dhruv Batra. Grad-CAM: Visual explanations from deep networks via gradient-based localization. *Proceedings of IEEE International Conference on Computer Vision*, pages 618–626, 2017.

[5] Michael W Sjoding, Daniel Taylor, Jonathan Motyka, Elizabeth Lee, Ivan Co, Dru Claar, Jakob I McSparron, Sardar Ansari, Meeta Prasad Kerlin, John P Reilly, et al. Deep learning to detect acute respiratory distress syndrome on chest radiographs: a retrospective study with external validation. *The Lancet Digital Health*, 3(6):e340–e348, 2021.

[6] Robert H. Peter, J. J. Morris, and Henry D. Mcintosh. Relationship of fibrillatory waves and P waves in the electrocardiogram. *Circulation*, 33:599–606, 1966.

[7] Roy M John, Usha B Tedrow, Bruce A Koplan, Christine M Albert, Laurence M Epstein, Michael O Sweeney, Amy Leigh Miller, Gregory F Michaud, and William G Stevenson. Ventricular arrhythmias and sudden cardiac death. *The Lancet*, 380(9852):1520–1529, 2012.

[8] John Hampton. *The ECG in practice*. Churchill Livingstone, 2003.

[9] Steven A Hicks, Jonas L Isaksen, Vajira Thambawita, Jonas Ghouse, Gustav Ahlberg, Allan Linneberg, Niels Grarup, Inga Strümke, Christina Ellervik, Morten Salling Olesen, et al. Explaining deep neural networks for knowledge discovery in electrocardiogram analysis. *Scientific Reports*, 11(1):1–11, 2021.

[10] Diederik P. Kingma and Jimmy Ba. Adam: A method for stochastic optimization. *3rd International Conference on Learning Representations, ICLR 2015, San Diego, CA, USA, May 7-9, 2015, Conference Track Proceedings*, 2015.

[11] David R Cox. Regression models and life-tables. *Journal of the Royal Statistical Society: Series B (Methodological)*, 34(2):187–202, 1972.

[12] Naomi DL Fisher and Gregory Curfman. Hypertension—a public health challenge of global proportions. *JAMA*, 320(17):1757–1759, 2018.

[13] Somayeh Sadeghi, Davood Khalili, Azra Ramezankhani, Mohammad Ali Mansournia, and Mahboubeh Parsaeian. Diabetes mellitus risk prediction in the presence of class imbalance using flexible machine learning methods. *BMC Medical Informatics and Decision Making*, 22(1):1–12, 2022.

[14] Afina S Glas, Jeroen G Lijmer, Martin H Prins, Gouke J Bonsel, and Patrick MM Bossuyt. The diagnostic odds ratio: a single indicator of test performance. *Journal of Clinical Epidemiology*, 56(11):1129–1135, 2003.

[15] Jacob Cohen. A coefficient of agreement for nominal scales. *Educational and Psychological Measurement*, 20(1):37–46, 1960.

[16] Valentin Amrhein, Sander Greenland, and Blake McShane. Scientists rise up against statistical significance. *Nature*, 567(7748):305–307, 2019.

[17] Borys Surawicz and Timothy Knilans. *Chou's electrocardiography in clinical practice: adult and pediatric*. Elsevier Health Sciences, 2008.

[18] Quinn McNemar. Note on the sampling error of the difference between correlated proportions or percentages. *Psychometrika*, 12(2):153–157, 1947.

[19] Maya Varma, Mandy Lu, Rachel Gardner, Jared Dunnmon, Nishith Khandwala, Pranav Rajpurkar, Jin Long, Christopher Beaulieu, Katie Shpanskaya, Li Fei-Fei, et al. Automated abnormality detection in lower extremity radiographs using deep learning. *Nature Machine Intelligence*, 1(12):578–583, 2019.

[20] Gari D Clifford, Chengyu Liu, Benjamin Moody, H Lehman Li-wei, Ikaro Silva, Qiao Li, AE Johnson, and Roger G Mark. AF classification from a short single lead ECG recording: The PhysioNet/computing in cardiology challenge 2017. *2017 Computing in Cardiology (CinC)*, pages 1–4, 2017.

[21] Shreyasi Datta, Chetanya Puri, Ayan Mukherjee, Rohan Banerjee, Anirban Dutta Choudhury, Rituraj Singh, Arijit Ukil, Soma Bandyopadhyay, Arpan Pal, and Sundeep Khandelwal. Identifying normal, AF and other abnormal ECG rhythms using a cascaded binary classifier. *2017 Computing in Cardiology (CinC)*, pages 1–4, 2017.
